# Radiogenic heating sustains long-lived volcanism and magnetic dynamos in super-Earths

**DOI:** 10.1126/sciadv.ado7603

**Published:** 2024-09-13

**Authors:** Haiyang Luo, Joseph G. O’Rourke, Jie Deng

**Affiliations:** ^1^ Department of Geosciences, Princeton University, Princeton, NJ, USA.; ^2^ School of Earth and Space Exploration, Arizona State University, Tempe, AZ, USA.

## Abstract

Radiogenic heat production is fundamental to the energy budget of planets. Roughly half of the heat that Earth loses through its surface today comes from the three long-lived, heat-producing elements (potassium, thorium, and uranium). These three elements have long been believed to be highly lithophile and thus concentrate in the mantle of rocky planets. However, our study shows that they all become siderophile under the pressure and temperature conditions relevant to the core formation of large rocky planets dubbed super-Earths. Mantle convection in super-Earths is then primarily driven by heating from the core rather than by a mix of internal heating and cooling from above as in Earth. Partitioning these sources of radiogenic heat into the core remarkably increases the core-mantle boundary (CMB) temperature and the total heat flow across the CMB in super-Earths. Consequently, super-Earths are likely to host long-lived volcanism and strong magnetic dynamos.

## Teaser

Entrainment of heat-producing elements in super-Earths’ cores produces intense, long-lasting volcanism and strong magnetic fields.

## INTRODUCTION

With more than 5000 confirmed exoplanets, including the commonly found super-Earths that are ~1 to 5 times the mass of Earth and possibly have core-mantle structures like Earth’s (*1*–*3*), the pursuit of Earth-like habitable planets (*4*–*6*) beyond our solar system remains a compelling subject of exploration. Two critical factors that determine a planet’s habitability are mantle degassing via volcanism, which can support a global volatile cycle necessary to maintain surface habitability (*7*, *8*), and the magnetic field, which can shield the planet’s atmosphere from the solar wind of its host star and protect its surface from high-energy charged particles (*9*). Active volcanism requires partial melting of mantle rock. Magnetic fields, using Earth as an example, are maintained by vigorous convection in the outer core, known as the geodynamo, and are governed by the heat flow across the core-mantle boundary (CMB) (*10*). The generation and sustenance of volcanism (*11*–*15*) and magnetic fields (*16*–*21*) in super-Earths are under debate because constraining the thermal evolution of exoplanets remains challenging (*22*).

A fundamental factor determining the existence of volcanism and magnetic fields is radiogenic heat production, which contributes to the overall heat budget of a planet and profoundly affects the temperature and dynamical properties of the core and mantle (*23*). In the Earth’s present-day mantle, the heat budget consists of secular cooling, heat flow from the core, and radiogenic heat production from three long-lived elements: potassium (K), thorium (Th), and uranium (U). These heat-producing elements (HPEs) account for almost half of the Earth’s total surface heat flow (*24*). Today, roughly one-third of the total radiogenic heat production is sequestered in the crust and upper mantle (*24*) because K, Th, and U are incompatible in silicate melt and are thus extracted from the convecting mantle by crustal production. In comparison, radiogenic heating in the core is typically discounted because HPEs are assumed to be highly lithophile (rock loving). Studies of exoplanets have assumed that HPEs always remain lithophile, even in super-Earths of larger sizes. This assumption stems from experimental (*25*–*30*) and computational (*31*, *32*) studies that have only investigated element partitioning in relation to Earth. However, it is not clear whether HPEs are still lithophile under extreme pressure (up to ~1400 GPa) and temperature (up to ~14,000 K) conditions relevant to super-Earths. Previous studies (*30*, *33*, *34*) revealed that some lithophile elements (e.g., B, Mg, Cl, and K) become less lithophile (more siderophile and iron loving) as pressure and temperature increase. Whether this shift toward siderophile behavior persists up to ~10 times larger pressures at the conditions of super-Earths is unknown, given the induced possibly marked structural changes of metal and silicate. For example, in silicate, the coordination number of Si with respect to O may increase from about 6 to 8 and intrinsically disordered phase may emerge (*35*). The metal-silicate partition coefficients of some other lithophile elements (e.g., F and P) show no notable changes with increasing pressure (>~20 GPa) and temperature (*34*, *36*). Should HPEs become siderophile and present in substantial proportions within the core, the generation and evolution of volcanism and magnetic dynamos in super-Earths would deviate from predictions outlined in existing models.

In this study, we used ab initio molecular dynamics (AIMD) simulations and the thermodynamic integration method to calculate the partition coefficients of K, Th, and U between metal and silicate melt (tables S1 and S2). These three elements are assumed to exist nominally as K_2_O, ThO, ThO_2_, UO, and UO_2_. By determining the chemical potentials of the five species in metal and silicate melts (figs. S1 and S2) and calculating their differences, we obtained the partition coefficients (Materials and Methods). Initially, we compared the partition coefficients of K_2_O in Fe-MgSiO_3_ and Fe_5.4_O (Fe with 5.0 wt % O)–MgSiO_3_ systems and found that adding 5 wt % oxygen to the iron core does not change the K_2_O partitioning notably (fig. S3). We then calculated the partition coefficients of K_2_O, ThO, ThO_2_, UO, and UO_2_ between Fe_5.4_O and MgSiO_3_ at 50, 135, 500, and 1000 GPa, corresponding to 3500, 5000, 9000, and 13,000 K, respectively. The assumed equilibration temperatures are near the estimated silicate liquidus at those pressures (*37*) and guarantee the liquid state of all our simulations.

In super-Earths, core formation should occur in tandem with planetary accretion, which features metal-silicate equilibration over a wide range of pressure/temperature conditions (*37*). However, simplified, single-stage models can reproduce the size and density of Earth’s core plus the abundances of several moderately siderophile elements in the mantle (*38*–*40*). Multistage models are more realistic (*38*, *41*, *42*), but the evolution of pressure, temperature, and redox conditions during accretion is difficult (perhaps impossible) to determine for exoplanets. Applied to Earth, the best-fit pressure/temperature conditions are ~55 GPa and ~3400 K in single-stage models (*38*, *40*, *43*), that is, near the silicate liquidus temperature at mid-mantle pressures. Single-stage models may likewise predict the abundances of HPEs in the cores and mantles of exoplanets. Here, we assume equilibration pressures of 50, 135, and 500 GPa for super-Earths with masses of ~1, 2 to 3, and 4 to 6 Earth masses (*M*_E_), respectively, because these pressures are representative of the middle-to-lower mantles of such exoplanets, which may melt during accretion even in the absence of late giant impacts (*20*, *44*, *45*). Increasing pressure and temperature may lead to more Mg, Si, and O atoms dissolved into the liquid metal and more Fe present in the silicate melt. Fe_5.4_O and MgSiO_3_ are used to represent the liquid metal and silicate melt, as we found that varying the compositions of liquid metal and silicate melt in a wide range (e.g., from Fe_54_O_11_K_2_ to Fe_72_Mg_3_Si_16_O_16_K_1_ and from MgSiO_3_ to Ca_2_Mg_30_Fe_4_Al_2_Si_24_O_87_) does not notably change the characteristics of the local structure of K/Th/U with respect to Fe/O (figs. S4 and S5). We performed two-phase molecular dynamics simulations of K-bearing metal-silicate coexistence (fig. S6). The consistency in the results between two-phase simulations and thermodynamic integration also indicates that Fe_5.4_O and MgSiO_3_ are acceptable choices for simplifying the metal and silicate compositions, respectively, when calculating the partition coefficients of K, Th, and U. In addition, the calculated chemical potentials of K_2_O and UO with more complex compositions (Fe_54_Mg_1_Si_3_O_10_ and Ca_2_Mg_30_Fe_4_Al_2_Si_24_O_87_) are comparable to those in Fe_5.4_O and MgSiO_3_ (tables S3 and S4).

## RESULTS AND DISCUSSION

**Fig. 1.**
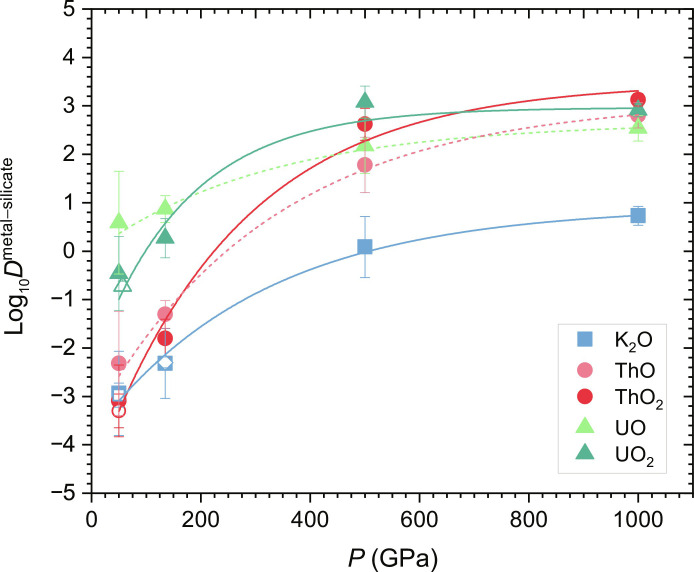
Partition coefficients of K, Th, and U. Calculated partition coefficients of K_2_O, ThO, ThO_2_, UO, and UO_2_ between metal (Fe with 5.0 wt % O) and silicate (MgSiO_3_) melts as a function of pressure (*P*). Partition coefficients are calculated using mole fractions. Solid symbols are our computational results. The corresponding open symbols represent experimental data (*30*) obtained at relatively low oxygen fugacity (IW–2, corresponding to 2 log(*f*O_2_) units below the iron-wüstite buffer). The open diamond symbol is the previous computational result of K_2_O (*31*). The experimental data acquired under lower pressures (~1 to 6 GPa) are not shown here, as the notable structural change of silicate melt within this pressure range may influence the partitioning behavior of elements in a manner that differs from the trend observed at higher pressures (*103*). Lines connecting computational results are best fit for K_2_O (blue solid), ThO (light red dashed), ThO_2_ (red solid), UO (light green dashed), and UO_2_ (dark green solid).

The metal-silicate partition coefficients that we calculated for K_2_O, ThO_2_, and UO_2_ at 50 GPa are in good agreement with experimental data (Fig. 1) (*30*), validating the compositions of metal and silicate that we adopted as well. The experiments suggested that U likely exists in the 2+ oxidation state in silicate melts around 50 GPa. This inferred oxidation state assumes that U is reduced to a zero-oxidation state in liquid iron. However, we find that U still strongly interacts with oxygen in liquid iron alloys and is likely in the 2+ oxidation state in liquid iron alloys according to Bader charge analysis (table S5). The electron localization functions (fig. S7) and projected densities of states (figs. S8 and S9) also reveal that U and Th strongly interact with oxygen in liquid iron alloys at 50 GPa (to a less extent for K), although these interactions weaken with pressure. As pressure rises, we observe an upward trend in the partition coefficients of the three heat producing elements, indicating a greater affinity for the metal phase. It is important to note that the degree of increase varies between the elements. Specifically, the metal-silicate partition coefficient of K_2_O increases from $0.0012_{-0.0010}^{+0.0073}$ at 50 GPa to $5.4_{-1.9}^{+3.0}$ at 1000 GPa. In comparison, the corresponding partition coefficients of ThO_2_ and UO_2_ rise from $0.00081_{-0.00066}^{+0.00367}$ to $1337_{-346}^{+466}$ and from $0.35_{-0.29}^{+1.66}$ to $813_{-213}^{+289}$ , respectively. Although the Bader charges of Th and U are found to decrease with increasing pressure (table S5), implying that the cases of ThO and UO may be more realistic at 500 and 1000 GPa, the differences in the partition coefficients between ThO_2_ and ThO (or UO_2_ and UO) are relatively small (~0.5 log unit). On the basis of our results, we estimated the initial distributions of the heat-producing radionuclides (^40^K, ^232^Th, ^235^U, and ^238^U) between core and mantle in super-Earths using the galactic chemical evolution model (*46*). Table 1 shows that the abundances of radionuclides in the core and mantle vary markedly with the pressure and temperature conditions of core-mantle differentiation, as a result of the change in their partitioning behaviors. For example, when ^40^K transitions from a lithophile element at 135 GPa (e.g., at ~2 to 3 *M*_E_) to a siderophile element at 500 GPa (~4 *M*_E_), its abundance in the core increases from 2.2 to 359 parts per billion. Note that the abundances of ^232^Th, ^235^U, and ^238^U remain relatively stable between 500 and 1000 GPa (e.g., ≥4 *M_E_*), because their partition coefficients at 500 GPa are already large enough to retain most of these radionuclides in the core.

**Table 1.** Distribution of radioactive heat-producing nuclides in super-Earths. ppb, parts per billion.

Nuclide abundances (ppb)	Pressure/temperature of core-mantle equilibration (GPa/K)			
	50/3500	135/5000	500/9000	1000/13,000
Mantle				
^40^K	463.3	462.4	292.1	128.9
^235^U	14.05	8.673	0.223	0.098
^238^U	53.42	32.97	0.849	0.374
^232^Th	154.9	153.8	5.174	0.501
Core				
^40^K	0.542	2.237	359.2	701.3
^235^U	4.917	16.19	33.90	34.16
^238^U	18.69	61.54	128.9	129.9
^232^Th	0.127	2.461	313.9	323.7

Our partition coefficients imply that super-Earths have markedly different heat budgets compared to Earth. We used parameterized models to determine how concentrating HPEs in the core affects planetary evolution (Materials and Methods). These models were not designed to reproduce individual planets. Instead, they illustrate the magnitudes and directions of effects on key processes, including near-surface melt production and dynamo activity. We modeled super-Earths with whole-mantle convection in the stagnant-lid regime and masses ≤6 *M*_E_ (fig. S4). Our models are not applicable for more massive super-Earths. Whole-mantle convection is less likely in super-Earths with masses above ~6 *M*_E_ as adiabatic compression leads to convective stability in the shallow mantle (*47*, *48*). Deep within super-Earths of >6 *M*_E_, silicates and metal may also become miscible at temperatures >10^4^ K, blurring the boundary between core and mantle. In each model, we tracked the potential temperature of the mantle (*T*_P_) and the temperature of the core (*T*_C_). We calculated the heat fluxes out of the mantle from conduction (*Q*_M,cond_) and melt production (*Q*_M,melt_). Using the initial abundances from Table 1, we computed the rates of heat production in the mantle (*H*_M_) and core (*H*_C_). Melt production sequesters HPEs from the mantle into the lithosphere (*H*_L_) in these models, which do not include a separate crust. The heat flux from the core (*Q*_C_) determines how fast the core cools, when the inner core nucleates, and whether convection is vigorous enough to generate a dynamo (*45*).

**Fig. 2.**
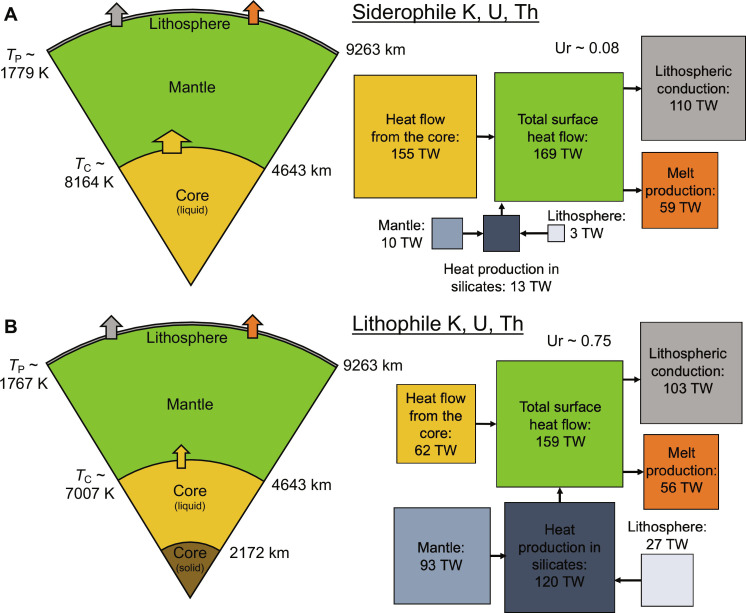
Thermal evolution of super-Earths. Snapshots of the thermal evolution of super-Earths (4 *M*_E_) after 4.5 billion years (Gyr). Cartoons on the left show the internal structure with the major layers to scale. Diagrams on the right show the global heat flow balance with the area of each square scaled to the amount of heat flux or production. HPEs are (**A**) partitioned between the mantle and core assuming metal-silicate equilibration at 500 GPa and 9000 K or (**B**) assumed to remain only in the silicates. Both models start with the same initial thermal state and use the lower-bound formula for the viscosity of post-perovskite (Materials and Methods).

Figure 2 shows snapshots of the evolution of a 4-*M*_E_ super-Earth at 4.5 billion years (Gyr) in our models. Both models start with the same initial temperatures and total amounts of internal heating. Figure 2A is the result of the partition coefficients from this study at 500 GPa and 9000 K, while Fig. 2B represents the conventional assumption that HPEs are lithophile. Our results imply that heat from the core drives mantle convection in super-Earths, with a near-zero Urey ratio (*49*). In contrast, the conventional model implies that internal heating is the most important heat source for mantle convection, with Urey ratios >0.50. Because the lithospheric viscosity is strongly temperature dependent, the mantle self-regulates in either case to a potential temperature of ~1800 K at 4.5 Gyr with ~140 to 150 km^3^ of melt production per year near the surface. If *H*_C_ ~0, then our models predict that the inner core grows to >2100 km by 4.5 Gyr. However, sequestering HPEs into the core makes the core hotter by >1000 K at 4.5 Gyr. Because radiogenic heating and inner core growth trade-off in the dissipation budget for the dynamo (*45*), both models predict a (dominantly dipolar) dynamo with a field strength of ~60 to 65 mT at the equator. However, the model with siderophile HPEs has relatively longer-lived magmatic (by ~1 Gyr or >13%) and magnetic activity (by ~8 Gyr or >87%).

**Fig. 3.**
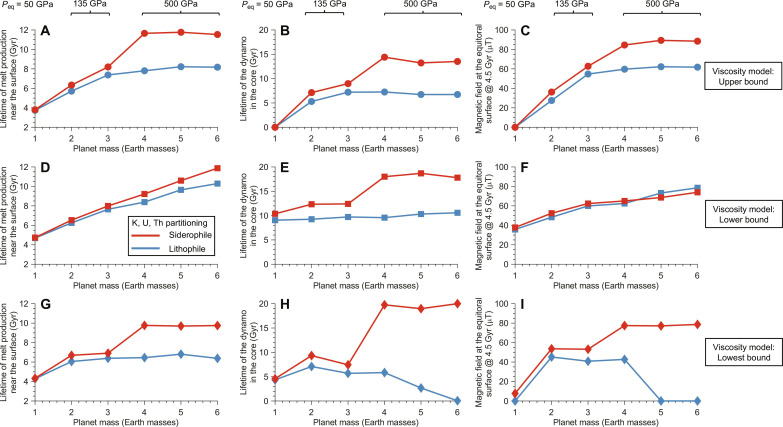
Volcanism and dynamos in super-Earths. Lifetimes of volcanism and dynamos in super-Earths in our models, which changed on the basis of the siderophile versus lithophile behavior of HPEs. We simulated the thermal evolution of terrestrial planets, with total masses of 1 to 6 *M*_E_, in the stagnant-lid regime for 20 Gyr (see Materials and Methods). In each subplot, red symbols represent models that assumed siderophile behavior for HPEs, using the equilibrium pressures listed at the top of the figure. Blue symbols represent models where the HPEs remained lithophile. Lines are drawn between symbols for illustration purposes only. From top to bottom, each row shows the output from models that used the (**A** to **C**) upper, (**D** to **F**) lower, and (**G** to **I**) lowest bound for the rheology of the lower mantle (see Materials and Methods), respectively. From left to right, each column shows [(A), (D), and (G)] when melt production near the surface ceased forever, [(B), (E), and (H)] when fluid motion in the core stopped, and [(C), (F), and (I)] the strength of the dipolar component at the surface after 4.5 Gyr.

Beyond the fundamental change on how mantle convection is powered, our results indicate that super-Earths can sustain volcanism and magnetic fields for much longer timespans than previously believed (*18*, *45*). Figure 3 illustrates the calculated lifetimes of melt production and dynamos for stagnant-lid planets with masses of 1 to 6 *M*_E_. Here, we used three formulations for the viscosity of the lower mantle that were based on studies of post-perovskite (*50*, *51*), the mineral phase that likely dominates the lower mantles of super-Earths of <4 *M*_E_. Other phases likely dominate the lower mantles of more massive super-Earths. Because the properties of super-Earths are uncertain, we tested the effects of varying viscosity by over ~10 orders of magnitude, which did not change our primary conclusions. As predicted by previous studies (*18*, *45*), we find that larger planets can produce longer-lived volcanism and magnetism, basically because of the cubic versus square scaling of volume and surface area with planetary radius. Lower viscosities in the basal mantle cause higher heat flow from the core, as expected, in turn, leading to more volcanism and stronger dynamos. Critically, our result is that partitioning HPEs into the core keeps the core hotter over geologic time while also maintaining a high heat flux into the basal mantle. Depending on the viscosity of the lower mantle, HPEs in the cores of >4-*M*_E_ super-Earths can increase, by >50 to 100%, the durations of near-surface melting and magnetism. We simulated planets with surface temperatures of ~400 K, too hot to stabilize liquid water. Regardless of the surface conditions, however, the siderophile behavior of HPEs should produce longer-lived volcanism and magnetic activity in models, relative to models that assume lithophile behavior. No matter the regime of mantle dynamics, HPEs in the core are never sequestered into the crust, so they remain available to power convection in the deep interior over geologic time. For example, imposing plate tectonics versus stagnant-lid convection would change the predicted lifetimes of mantle melting and core convection, but implementing the siderophile behavior of HPEs should have the same directional effect (i.e., increasing those lifetimes). Our study thus implies that super-Earths are likely to remain geologically active for at least the main-sequence lifetimes of Sun-like stars.

Ultimately, our work shows that the distribution of HPEs within a planet is as important to planetary evolution as the bulk inventory of those elements. Their partitioning behavior is also as important in models as other factors that have received much attention, such as the viscosity of the lower mantle and the thermal conductivity of the core (discussed in Materials and Methods). Recent work emphasizes that exoplanet-hosting stars have concentrations of K, Th, and U that vary by factors of a few relative to those in Earth (*52*). We find that metal-silicate partitioning during core formation can change the abundances of HPEs in the mantle by at least that amount. Previous studies (*16*, *52*) also predicted that planets with more internal heating are less likely to host dynamos. We conclude the opposite for super-Earths because we find that such heating is concentrated in the core, where it increases the core heat flux, rather than in the mantle, which would decrease the core heat flux. Although our models here are only parameterized and neglect several processes (discussed in Materials and Methods), they clearly show that the siderophile behavior of HPEs transforms the heat budgets of super-Earths. Future models of individual worlds should consider the core as the furnace of planetary evolution.

## MATERIALS AND METHODS

### Chemical potentials

We first used the method described in the study on water partitioning (*53*) to calculate the chemical potentials of K_2_O, ThO_2_, and UO in metal (Fe and Fe_5.4_O)/silicate (MgSiO_3_) melt. The Gibbs free energy of a system under defined pressure (*P*), temperature (*T*), and solute concentration (*x*) can be expressed as

$$G\left(p,T,x\right)=\bar{G}\left(p,T,x\right)-TS_{\text{mix}}^{\text{system}}$$ (1)

where $\bar{G}\left(p,T,x\right)$ is the excess free energy and $S_{\text{mix}}^{\text{system}}$ is the ideal gas mixing entropy. Then, the chemical potential of K_2_O/ThO_2_/UO can be written as

$$\mu_{K_{2}O/{\text{ThO}}_{2}/\text{UO}}\left(p,T,x\right)={\bar{\mu }}_{K_{2}O/{\text{ThO}}_{2}/\text{UO}}\left(p,T,x\right)-TS_{\text{mix}}^{K_{2}O/{\text{ThO}}_{2}/\text{UO}}$$ (2)

Here, ${\bar{\mu }}_{K_{2}O/{\text{ThO}}_{2}/\text{UO}}\left(p,T,x\right)$ and $S_{\text{mix}}^{K_{2}O/{\text{ThO}}_{2}/\text{UO}}$ can be determined using

$${\bar{\mu }}_{K_{2}O/{\text{ThO}}_{2}/\text{UO}}\left(p,T,x\right)=\bar{G}\left(p,T,x\right)+\left(1-x\right)\frac{\partial \bar{G}\left(p,T,x\right)}{\partial x}$$ (3)

$$S_{\text{mix}}^{K_{2}O/{\text{ThO}}_{2}/\text{UO}}=S_{\text{mix}}^{\text{system}}+\left(1-x\right)\frac{\partial S_{\text{mix}}^{\text{system}}}{\partial x}$$ (4)

The variations of $\bar{G}\left(p,T,x\right)$ per formula with respect to *x* (figs. S1 and S2) for our seven systems—Fe_1−*x*_(K_2_O)*_x_*, (Fe_5.4_O)_1−*x*_(K_2_O)*_x_*, (MgSiO_3_)_1−*x*_(K_2_O)*_x_*, (Fe_5.4_O)_1−*x*_(ThO_2_)*_x_*, (MgSiO_3_)_1−*x*_(ThO_2_)*_x_*, (Fe_5.4_O)_1−*x*_(UO)*_x_*, and (MgSiO_3_)_1−*x*_(UO)*_x_*—all exhibited a good fit when modeled with a linear equation

$$\bar{G}\left(p,T,x\right)=a+\mathrm{bx}$$ (5)

where *a* and *b* are the fitting parameters. Consequently, ${\bar{\mu }}_{K_{2}O/{\text{ThO}}_{2}/\text{UO}}$ $\left(p,T,x\right)$ can be determined as *a* + *b*, and the uncertainties of ${\bar{\mu }}_{K_{2}O/{\text{ThO}}_{2}/\text{UO}}\left(p,T,x\right)$ can be obtained through SE propagation considering the uncertainties of *a* and *b* determined from the linear fitting.

The $S_{\text{mix}}^{K_{2}O/{\text{ThO}}_{2}/\text{UO}}$ for the seven systems, computed using Eq. 4, are as follows

$$S_{\text{mix}}^{K_{2}O\;\text{in}\;\text{Fe}}=-k_{B}\left(2\text{ln}\frac{2x}{1+2x}+\text{ln}\frac{x}{1+2x}\right)$$ (6)

$$S_{\text{mix}}^{K_{2}O\;\text{in}\;{\text{Fe}}_{5.4}O}=-k_{B}\left(2\text{ln}\frac{2x}{6.4-3.4x}+\text{ln}\frac{1}{6.4-3.4x}\right)$$ (7)

$$S_{\text{mix}}^{K_{2}O\;\text{in}\;{\text{MgSiO}}_{3}}=-k_{B}\left(2\text{ln}\frac{2x}{5-2x}+\text{ln}\frac{3-2x}{5-2x}\right)$$ (8)

$$S_{\text{mix}}^{{\text{ThO}}_{2}\;\text{in}\;{\text{Fe}}_{5.4}O}=-k_{B}\left(\text{ln}\frac{x}{6.4-3.4x}+2\text{ln}\frac{1+x}{6.4-3.4x}\right)$$ (9)

$$S_{\text{mix}}^{{\text{ThO}}_{2}\;\text{in}\;{\text{MgSiO}}_{3}}=-k_{B}\left(\text{ln}\frac{x}{5-2x}+2\;\text{ln}\frac{3-x}{5-2x}\right)$$ (10)

$$S_{\text{mix}}^{\text{UO}\;\text{in}\;{\text{Fe}}_{5.4}O}=-k_{B}\left(\text{ln}\frac{x}{6.4-4.4x}+\text{ln}\frac{1}{6.4-4.4x}\right)$$ (11)

$$S_{\text{mix}}^{\text{UO}\;\text{in}\;{\text{MgSiO}}_{3}}=-k_{B}\left(\text{ln}\frac{x}{5-3x}+\text{ln}\frac{3-2x}{5-3x}\right)$$ (12)

Then, at equilibrium when $\mu_{K_{2}O/{\text{ThO}}_{2}/\text{UO}}^{{\text{Fe}}_{5.4}O}\left(p,T,x\right)=\mu_{K_{2}O/{\text{ThO}}_{2}/\text{UO}}^{{\text{MgSiO}}_{3}}\left(p,T,y\right)$, the partition coefficients that we need, $D_{K_{2}O/{\text{ThO}}_{2}/\text{UO}}^{{\text{Fe}}_{5.4}O-{\text{MgSiO}}_{3}}=x/y$ , can be readily obtained using Eq. 2, and the associated errors are propagated from the uncertainties of the chemical potentials. We calculated the chemical potentials of ThO and UO_2_ by adopting alchemical free energy method. For instance, the Th atom in the ThO_2_-bearing metal or silicate melt was converted to U atom to obtain the chemical potentials of UO_2_. More details of this method can be found in previous publications (*54*, *55*).

### Molecular dynamics simulations

We conducted simulations of the iron melt using 64 Fe atoms, the iron-alloy melt using 54 Fe and 10 O atoms, and the silicate melt using 32 formula units of MgSiO_3._ Subsequently, K_2_O, ThO_2_, and UO were introduced into the supercells. Born-Oppenheimer AIMD simulations were performed using the projector augmented wave method (*56*, *57*) and PBEsol exchange-correlation functional (*58*), as implemented in Vienna Ab initio Simulation Package (*59*). Valence configurations for elements were specified as follows: Fe 3p^6^3d^7^4s^1^, Mg 2p^6^3s^2^, Si 3s^2^3p^2^, O 2s^2^2p^4^, K 3s^2^3p^6^4s^1^, Th 6s^2^6p^6^6d^2^7s^2^, and U 6s^2^6p^6^5f ^3^6d^1^7s^2^. Previous studies (*60*–*65*) tested these PAW potentials by comparing with all-electron calculations and found good agreement up to the terapascal level. Thermal equilibrium between ions and electrons was maintained using the Mermin functional (*66*). We tested the relativistic effect for heavy elements Th and U by doing spin orbit coupling calculations and found that the relativistic effect behaves similarly in metal and silicate phases, resulting in no significant change in the calculated partition coefficients. We evaluated the magnetic states of Th and U in silicate melt and Fe, Th, and U in metal melt using spin-polarized calculations. We found that all atoms remain in a nonmagnetic state when the pressure exceeds 135 GPa. Only U in silicate melt and Fe in metal melt exhibit a small magnetic moment (<0.75 μB) at 50 GPa. Previous studies indicate that ignoring magnetism does not significantly affect metal-silicate partitioning of elements such as H (*53*), noble gas (*67*), and Pu (actinide element like Th and U) (*68*). The agreement between our calculated partition coefficients and experimental data at 50 GPa supports this approximation, especially considering our main focus is on super-Earth scenarios. Thus, all our simulations were performed without spin polarization. Each simulation was run for up to 20,000 steps with a timestep of 1.0 fs. Brillouin zone sampling was done at the gamma point only, with a plane-wave energy cutoff set to 600 and 800 eV for liquid iron/iron-alloy and silicate melts, respectively. For Th/U-bearing silicate melts, a plane-wave energy cutoff of 680 eV was used. To correct for pressure in iron/iron-alloy, a 2 × 2 × 2 Monkhorst-Pack mesh of *k*-points was used because of the relatively small system size. The effect of the *k*-point mesh on the calculated chemical potentials is negligible (table S6). We corrected the difference in the energy cutoff between silicate melts and Th/U-bearing silicate melts with thermodynamic perturbation using the second-order cumulant expansion. We used a NVT canonical ensemble (i.e., number of atoms, volume, and temperature remain constant during simulation) with temperature regulation achieved through a Nosé-Hoover thermostat (*69*). Equilibrium volume was determined by conducting several NVT simulations at different volumes around the target pressure and fitting the resulting pressure-volume data. Radial distribution function and mean square displacement analyses were conducted to ensure the liquid state of our simulated systems.

### Thermodynamic integration

We computed the Gibbs free energy of iron/iron-alloy and silicate melts through thermodynamic integration from the ideal gas state (*70*). This involved integrating the difference in internal energy between the melt (*U*_1_) and the ideal gas (*U*_0_) with respect to the coupling parameter λ, as follows

$$\Delta G_{0-1}=\int_{0}^{1}d\lambda {\langle U_{1}\left(R\right)-U_{0}\left(R\right)\rangle }_{\lambda }$$ (13)

To address the numerical instabilities arising from the proximity of noninteracting atoms at λ = 0, we used the variable transformation approach (*70*) with $\lambda \left(x\right)={\left(\frac{x+1}{2}\right)}^{1/\left(1-k\right)}$ , leading to

$$\Delta G=\frac{1}{2\left(1-\kappa \right)}\int_{-1}^{1}f\left[\lambda \left(x\right)\right]\lambda {\left(x\right)}^{k}\mathrm{dx}$$ (14)

where we chose *k* = 0.8. An eight-point Gauss-Lobatto rule was used to compute this integral. The errors in internal energies were determined using the blocking average method (*71*). The errors of the numerically integrated free energies were derived through SE propagation, i.e., for a numerical integral *I* = ∑*_i_*ω*_i_ f_i_*, $\sigma^{2}\left(I\right)=\sum_{i}\omega_{i}^{2}\sigma^{2}\left(f_{i}\right)$ , where σ(*A*) represents the error for the quantity *A*. We also tested using Weeks-Chandler-Andersen fluid rather than the simple ideal gas as the reference state of liquid and found the difference in free energies is negligible. More sophisticated integrated pathways are unlikely to change significantly our partitioning results as they only result in difference in free energy at tens of milli–electron volts per atom (*72*), which is around one order of magnitude smaller than the error bar of chemical potential (table S2).

The Gibbs free energy of the ideal gas was computed as

$$G^{\mathrm{ig}}=F^{\mathrm{ig}}+\mathrm{PV}=-k_{B}T\sum_{i}\text{ln}\left(\frac{V^{N_{i}}}{N_{i}!{\Lambda_{i}}^{3N_{i}}}\right)+\mathrm{PV}$$ (15)

Here, *k*_B_ represents the Boltzmann constant, *T* denotes temperature, *P* stands for pressure, *V* represents volume, *N_i_* signifies the number of atoms for element *i*, Λ*_i_* denotes the thermal wavelength of element *i* given by Λ*_i_* = *h*/(2π*M_i_k*_B_*T*)^1/2^, with *h* representing the Plank’s constant and *M_i_* representing the atomic mass of element *i*. All calculations were calibrated to the target pressures adopting the thermodynamic relation

$$G\left(P_{2},T\right)-G\left(P_{1},T\right)={\left(\int_{P_{1}}^{P_{2}}\mathrm{VdP}\right)}_{T}$$ (16)

### Thermal evolution models

We used parameterized models for the thermal evolution of terrestrial exoplanets. These models assume that the silicate mantle is fully solid, while the metallic core is fully molten. In the immediate aftermath of planetary accretion, the silicate mantle should be at least partially molten. Prior studies show that a magma ocean in the mantle could solidify from the bottom upward in ~10 to 100 million years (Myr) (*73*). Here, our models start after this solidification is complete, however long it takes. In other words, *t* in our models is the time after the mantle has fully solidified, which is less than the time after the planet has accreted. We chose not to model planetary accretion in detail because our focus is on the long-term evolution of terrestrial planets.

Our models also do not yet include a basal magma ocean (BMO). As discussed below, a large reservoir of molten silicates could survive in the lower mantle if the mantle crystallizes from the middle outward. This BMO could take billions of years to solidify, because the overlying, solid mantle is insulating and the adjacent core is a heat sink (*74*). Adding a BMO to our models is a priority but will introduce additional complexity. We chose to start with models that assume a fully solid mantle to set a foundation for future models that include a BMO.

In our models, we first specify the radial structure and thermophysical properties of each planet. We follow (*45*) in assuming that the core constitutes 32.5% of the total planetary mass, as for Earth. To isolate the effects of individual parameters in our models, we need to assume that every planet operates with the same mode of mantle convection. We chose to model stagnant-lid convection, which is perhaps the most natural (albeit not the most Earth-like) mode for terrestrial planets. For simplicity, we adapted the scaling laws for stagnant-lid convection from (*8*) and the references therein, unless otherwise cited. However, we augmented their approach to include the heat flux from the core in the mantle’s heat budget and to track the evolution of the core in detail to better predict when a dynamo would exist. We also changed the treatment of mantle viscosity to use the results of (*50*) and (*51*). Last, we simplified the description of melt production to enable more straightforward comparisons between models.

In this section, we first describe the governing equations for our model. Next, we explain how we run an individual model. Then, we present all the models that we ran for this study. Last, we discuss some limitations of our models and priorities for future work.

#### Governing equations

Figure S10 is a cartoon of the internal structure, labeling the properties that we track over time. The temperature of the mantle changes with time (*t*) according to its energy budget

$$V_{M}\rho_{M}C_{M}\frac{dT_{M}}{\mathrm{dt}}=H_{M}+Q_{C}-Q_{M}$$ (17)

where *V*_M_, ρ_M_, and *C*_M_ = 1250 J kg^−1^ K^−1^ are the volume, average density, and specific heat capacity of the mantle, respectively. Here, *T*_M_ is the potential temperature of the mantle (i.e., the projection of the adiabatic thermal gradient in the convective mantle to the planet’s surface). The total amount of radiogenic heating in the convective mantle is *H*_M_, which does not include any contribution from HPEs in the lithosphere. The total heat flux from the core into the mantle is *Q*_C_. We assume that the total mantle heat flux (*Q*_M_) is split into two parts: heat conducted into the stagnant lid (*Q*_M,cond_) and heat associated with the production and transport of melt (*Q*_M,melt_).

The viscosity of the solid silicates governs the rate at which the mantle and core can lose heat. We assume that the mantle viscosity changes with temperature and pressure as

$$\eta \left(T,P\right)=\eta_{0}\text{exp}\left(\frac{H}{\mathrm{RT}}-\frac{E}{RT_{0}}\right)$$ (18)

where η_0_ is a reference viscosity at a reference temperature (*T*_0_ = 1600 K) and *R* is the universal gas constant (*50*). Here, *E* is an activation energy. The effective enthalpy, *H* = *E* + *PV*, depends on the effective activation volume, *V* = *V*_0_exp(–*P*/*P*_decay_). To calculate the viscosity of the upper mantle, we take *E* = 300 kJ mol^−1^, *P* = 0, and η_0_ = 10^20^ Pa·s (*50*). For the lower mantle, we test three sets of values that reflect the current uncertainty on the rheology of post-perovskite (*50*). We use the “lower bound” and “upper bound” from table 2 of (*50*). The lower bound has *E* = 162 kJ mol^−1^, *V*_0_ = 1.4 × 10^−6^ m^3^ mol^−1^, *P*_decay_ = 1610 GPa, and η_0_ = 1.9 × 10^21^ Pa·s, while the upper bound features *E* = 780 kJ mol^−1^, *V*_0_ = 1.7 × 10^−6^ m^3^ mol^−1^, *P*_decay_ = 1100 GPa, and η_0_ = 1.05 × 10^34^ Pa·s. As expressed by their names, the lower-bound rheology always predicts a less viscous lower mantle than the upper-bound rheology. However, the upper-bound rheology implies that the lower mantle viscosity is relatively insensitive to planetary mass; the effects of increasing pressure and temperature in larger planets balance each other out. In contrast, the lower-bound rheology predicts that massive planets have more viscous lower mantles (e.g., by ~6 orders of magnitude for ~6-*M*_E_ versus ~1-*M*_E_ planets). Last, Karato (*51*) argued that lower mantle viscosity could decrease with increasing planetary mass, perhaps by ~2 orders of magnitude up to ~6 *M*_E_. To produce this behavior, our “lowest-bound” viscosity model uses *E* = 650 kJ mol^−1^, *V* = 0.2 × 10^−6^ m^3^ mol^−1^ (i.e., infinite *P*_decay_), and η_0_ = 10^33^ Pa·s. Figure S11 shows the lower mantle viscosity predicted by all three models, when *T*_M_ = 1600 K, as a function of pressure at the CMB.

Convective vigor in the mantle increases with its temperature. At each timestep in our models, we first calculate the Frank-Kamenetskii parameter

$$\Theta_{\text{FK}}=\frac{E\left(T_{M}-T_{S}\right)}{RT_{M}^{2}}$$ (19)

where we set the surface temperature to *T*_S_ = 400 K always. Varying *T*_S_ by up to a few hundred kelvins would obviously imply hugely different surface conditions but would not substantially change the thermal evolution of the mantle and the core, if we continued to assume that the mantle operated in the stagnant-lid regime. Therefore, we can illustrate the main effects of the siderophile behavior of HPEs using models with a fixed *T*_S_. Next, we compute the Rayleigh number of the mantle

$$\text{Ra}=\frac{\rho_{M}g\alpha_{M}\left(T_{M}-T_{S}\right){\left(R_{P}-R_{C}\right)}^{3}}{\kappa_{M}\eta }$$ (20)

where *g* is the gravitational acceleration at the surface; α_M_ = 10^−5^ K^−1^ and κ_M_ = 10^−6^ m^2^ s^−1^ are the thermal expansivity and thermal diffusivity of the mantle, respectively; and *R*_P_ and *R*_C_ are the radii of the planet and core (table S7), respectively. We then compute a characteristic velocity for convection as in (*8*), based on “scaling *I*” for linear viscosity with a stress exponent of *n* = 1 from (*75*)

$$v=\frac{0.05\kappa_{M}}{R_{P}-R_{C}}{\left(\frac{\text{Ra}}{\Theta_{\text{FK}}}\right)}^{\frac{2}{3}}$$ (21)

In the stagnant-lid regime, the temperature contrast across the convective boundary layer depends on the temperature dependence of viscosity (*75*–*79*)

$$\Delta T_{\mathrm{rh}}\approx a_{\mathrm{rh}}\Theta_{\text{FK}}^{-1}\left(T_{M}-T_{S}\right)=\frac{a_{\mathrm{rh}}RT_{M}^{2}}{E}$$ (22)

where *α_rh_* is a constant. The mantle heat flux per unit area is then

$$F_{M}\approx 0.5k_{M}\Theta_{\text{FK}}^{-\frac{4}{3}}Ra^{\frac{1}{3}}\left(\frac{T_{M}-T_{S}}{R_{P}-R_{C}}\right)$$ (23)

where positive values indicate that heat is moving out of the mantle (*75*). Here, *k*_M_ ~ 5 W/(m·K) is the thermal conductivity of the upper mantle (*8*). Last, the mantle heat flux that is conducted into the stagnant lid is simply that flux multiplied by the surface area of the mantle: *Q*_M,cond_ = (4/3)*p*(*R*_P_ − δ*_u_*)^2^*F*_M_, where δ*_u_* is the thickness of the lithosphere.

We then use an energy balance to determine how the thickness of the lithosphere changes over time. The lid changes in response to the mantle heat flux (*8*, *80*, *81*)

$$\rho_{M}C_{M}\left(T_{M}-T_{B}\right)\frac{d\delta_{u}}{\mathrm{dt}}=-F_{M}-k_{M}{\left.\frac{\partial T}{\partial z}\right|}_{z=R_{P}-\delta_{u}}$$ (24)

The rightmost term is a boundary condition that depends on the amount and distribution of radiogenic heating in the lithosphere. Unterborn *et al.* (*8*) tracked the thicknesses of the crust and lithosphere separately, because the crust (a compositional layer) can be thinner than the lithosphere (a layer defined on the basis of its mechanical and thermal properties). In their models, HPEs are concentrated within the crust, whereas any lithosphere below the crust has the same concentration of HPEs as the rest of the mantle. Mantle convection tends to entrain and recycle any crust that becomes thicker than the lithosphere. For simplicity, we assume here that the HPEs are distributed uniformly throughout the lithosphere. We do not separately track the thickness of the crust or its inventory of HPEs. Because our study is not focused on the detailed structure of the crust and lithosphere, this simplification does not affect our main results. In any case, the temperature at the base of the lithosphere is

$$T_{B}=T_{M}-\Delta T_{\mathrm{rh}}=T_{M}\left(1-\frac{a_{\mathrm{rh}}RT_{M}}{E}\right)$$ (25)

where the rightmost term relates to how hot the silicates need to become to participate in mantle convection (*75*). We set α*_rh_* ~ 2.5 following (*8*), although Solomatov and Moresi (*75*) suggested that α*_rh_* ~ 2.4 for the viscosity scaling used in Eq. 21. This discrepancy does not affect our results. Last, the boundary condition used in Eq. 24 is

$$k_{M}{\left.\frac{\partial T}{\partial z}\right|}_{z=R_{P}-\delta_{u}}=0.5x_{u}\delta_{u}-\frac{k_{M}\left(T_{B}-T_{S}\right)}{\delta_{u}}$$ (26)

where *x_u_* is the volumetric rate of radiogenic heat production (i.e., in units of watts per cubic meter) in the lithosphere, i.e., the sum of individual contributions from K, Th, and U (*8*). This equation represents the steady-state thermal gradient within the lithosphere, where conductive heat transport balances radiogenic heating (*82*). As explained below, the lithosphere and mantle have different rates of radiogenic heat production.

Melt production removes heat from the mantle and sequesters HPEs in the lithosphere. We assume that melting begins below the lithosphere at a pressure that increases with *T*_M_

$$P_{i}=1\;\text{GPa}\left(\frac{T_{M}-1423\;K}{100\;K}\right)$$ (27)

based on a solidus for dry peridotite (*82*–*84*). The equivalent depth is *d*_melt_ = *P_i_*/(ρ_L_*g*), where ρ_L_ ~ 3300 kg m^−3^ is the average density of the lithosphere. Melting stops at the base of the lithosphere, at a final pressure of

$$P_{f}=\rho_{L}g\delta_{u}$$ (28)

The melt fraction depends on the difference between these two pressures

$$\phi =0.075\left(\frac{P_{i}-P_{f}}{1\;\text{GPa}}\right)$$ (29)

Following (*8*) and the references therein, the volumetric rate of melt production is

$$f_{m}=17.8\pi R_{P}v\left(d_{\text{melt}}-\delta_{u}\right)\phi$$ (30)

In this study, we do not estimate or compute the relative fraction of extrusive volcanism versus intrusive magmatism, simply the total rate of near-surface melting. The heat flux associated with melt production is then

$$Q_{M,\text{melt}}=f_{m}\rho_{M}\left(C_{M}\Delta T_{m}+L_{M}\right)$$ (31)

where Δ*T*_m_ = *T*_M_ − (6.6766 × 10^−4^ K m^−1^)*d*_melt_ − *T*_S_ is the difference between the temperature of the melt at the base of the lithosphere versus at the surface (*8*). The latent heat of melting for the silicates is *L*_M_ = 600 kJ kg^−1^ (*8*). Last, HPEs are incompatible elements, so they partition into the lithosphere because of melt production. However, if the lithosphere thins (i.e., *d*δ*_u_*/*dt* < 0), then some HPEs are recycled back into the mantle. In each timestep, we track the change(s) in the lithospheric heat production for each isotope (*8*, *82*)

$$\begin{array}{c} \frac{dH_{L,i}}{\mathrm{dt}}=\frac{x_{m,i}f_{m}}{\phi }\left[1-{\left(1-\phi \right)}^{\frac{1}{D_{i}}}\right]\\ -x_{u,i}\left(f_{m}-4\pi {\left[R_{P}-\delta_{u}\right]}^{2}\text{min}\left[0,\frac{d\delta_{u}}{\mathrm{dt}}\right]\right)-\lambda_{i}H_{L,i} \end{array}$$ (32)

where *x*_m,*i*_ is the volumetric rate of heat production from an individual isotope in the mantle and λ*_i_* is the isotope-specific decay constant. The partition coefficients are *D_i_* = 0.0011, 0.0012, and 0.0029 for K, U, and Th, respectively. What is added to the crust is subtracted from the mantle, and vice versa (*8*, *82*)

$$\begin{array}{c} \frac{dH_{M,i}}{\mathrm{dt}}=-\frac{x_{m,i}f_{m}}{\phi }\left[1-{\left(1-\phi \right)}^{\frac{1}{D_{i}}}\right]\\ +x_{u,i}\left(f_{m}-4\pi {\left[R_{P}-\delta_{u}\right]}^{2}\text{min}\left[0,\frac{d\delta_{u}}{\mathrm{dt}}\right]\right)-\lambda_{i}H_{M,i} \end{array}$$ (33)

In general, sequestering HPEs in the crust leads to colder mantle temperatures over time and thus less melt production and a shorter lifetime of volcanism.

We use a boundary layer model to calculate the heat flux out of the core. That is, we assume that the steady-state thickness of the boundary layer results in a Rayleigh number close to the critical value of ~10^3^ for convection (i.e., the entire boundary layer can be mobile, unlike in the stagnant-lid atop the mantle). The thickness of that boundary layer above the core is then

$$\delta_{l}={\left[\frac{10^{3}\kappa_{l}\eta \left(0.5\left[T_{C}+T_{L}\right]\right)}{\rho_{l}g_{l}\alpha_{l}\left(T_{C}-T_{L}\right)}\right]}^{\frac{1}{3}}$$ (34)

where *T*_L_ and *T*_C_ are the temperatures of the basal mantle and the upper core, respectively (*45*). To calculate the temperature in the lower mantle, we assume that *T*_L_ = *T*_P_[ρ_l_/(4100 kg/m^3^)]*^G^*, where *G* ranges from ~1.11 to 1.01 (table S7). This expression reproduces the adiabat from figure 2 of (*50*). In the lower mantle, *k*_l_, ρ_l_, *g*_l_, and α_l_ are the local thermal diffusivity, density, gravitational acceleration, and thermal expansivity, respectively, all calculated as in (*45*). The total heat flow across the CMB is then

$$Q_{C}=4\pi R_{C}^{2}k_{l}\left(\frac{T_{C}-T_{L}}{\delta_{l}}\right)$$ (35)

Our approach assumes whole-mantle convection. In larger super-Earths, adiabatic compression may cause layered convection (*47*, *48*). Because our models do not include layered mantle convection, we studied only super-Earths up to a mass of 6 *M*_E_. However, larger super-Earths are rare in our galaxy. Observations have revealed that exoplanets larger than ~1.6 Earth radii (~4 to 5 *M*_E_) typically have thick volatile envelopes, meaning that they are not rocky (*72*). Statistics aside, missions such as the NASA Transiting Exoplanet Survey Satellite and follow-up observations will provide mass and radius measurements that can guide future models.

We model the core’s response to cooling exactly as in (*45*), which was heavily based on (*85*). The heat budget for the core is

$$Q_{C}=Q_{\text{RC}}+Q_{\text{SC}}+Q_{\text{PC}}+Q_{\text{GC}}+Q_{\text{LC}}+Q_{\text{IC}}$$ (36)

where the total heat flow is partitioned into six heat flow terms on the right side: radiogenic heat (RC), secular cooling (SC), precipitation of light elements near the core/mantle boundary (PC), gravitational energy associated with the exclusion of light elements from the inner core (GC), the latent heat of freezing for the inner core (LC), and heat conducted out of the inner core (IC), assuming that the inner core has effectively infinite thermal conductivity (*45*). We can write each of the terms, except *Q*_RC_, in the form *Q_i_* = Θ*_i_*(*dT*_C_/*dt*), where Θ*_i_* is a function only of the thermophysical parameters of the core (*45*). Thus, we calculate the cooling rate of the core (*45*)

$$\frac{dT_{C}}{\mathrm{dt}}=\frac{Q_{C}-Q_{R}}{\Theta_{\text{SC}}+\Theta_{\text{PC}}+\Theta_{\text{GC}}+\Theta_{\text{LC}}+\Theta_{\text{IC}}}$$ (37)

We use a fourth-order polynomial to parameterize the density profile of the core. Therefore, the equations for the individual heat flow terms are algebraically complex. Interested readers are encouraged to consult (*45*), for the complete details, including the definition of the liquidus in the core. Here, we calculate the thermal conductivity of the core as *k*_c_ = −35.64 W m^−1^ K^−1^ + (0.027 W m^−1^ K^−2^)*T_D_*, where *T_D_* is the average temperature in the liquid portion of the core. This fitted linear temperature dependence of the thermal conductivity is based on the computed results (*86*) corrected by electron-electron scattering (*87*) for the Fe_0.79_Si_0.08_O_0.13_ liquid using an adiabat of *T*_ICB_ = 5500 K. We assume that the super-Earths have Earth-like abundances of light elements, meaning that the solidification of the inner core releases gravitational energy. After the inner core nucleates, its growth rate (*dR_I_*/*dt*) is directly proportional to the core’s cooling rate (*dT_C_*/*dt*). Our models self-consistently capture how the flux of light elements from the inner core depresses the liquidus of the outer core.

We use the dissipation budget for the core to determine when a dynamo may exist (*45*, *85*). When positive dissipation (Φ > 0) is available, we use mixing length theory and the Coriolis-Inertial-Archimedes (CIA) and the magneto-Archimedes-Coriolis (MAC) force balances to estimate the strength of the dipole component of the magnetic field (*88*). Note that we do not use the same velocity scalings for the liquid core and the solid mantle because their viscosities are so different. For example, from mixing length theory, the strength of the magnetic field in the core is (*89*)

$$B_{C}={\left[2c\mu_{0}{\left(\rho_{0}R_{C}^{2}\Phi^{2}\right)}^{\frac{1}{3}}\right]}^{\frac{1}{2}}$$ (38)

where *c* ~ 0.63 is a constant of proportionality, ρ_0_ is the density at the center of the core, and μ_0_ is the permeability of free space. The surface field strength of the dipole component is then

$$B_{S}=\frac{1}{7}B_{C}{\left(\frac{R_{C}}{R_{P}}\right)}^{3}$$ (39)

where the prefactor of ^1^/_7_ represents an Earth-like power spectrum for the magnetic field (*89*). We report the magnetic field strength using the results from mixing length theory because they are closest to the strength of Earth’s magnetic field for a 1-*M*_E_ planet (i.e., within a factor of a few). Using the MAC or CIA scaling laws would return lower field strengths for all planets, but with the same effects from changing planetary mass and/or the partitioning behavior of HPEs.

In the core, there are two competing effects of radiogenic heating on the prospects for a dynamo. For a fixed amount of heat flow extracted into the mantle, more radiogenic heating in the core tends to decrease the dissipation available for a dynamo, because radiogenic heating is less efficient as a power source than secular cooling and the exclusion of light elements from the growing inner core (if existing) (*85*). However, we should not assume that the core heat flux stays fixed if the partitioning behavior of HPEs changes. Radiogenic heating also slows the cooling of the core. Partitioning radioactive elements into the core means that they are less abundant in the mantle, implying that the heat flow across the CMB should be relatively higher, which would increase the available dissipation. Our models revealed that this second effect is more important, meaning that adding HPEs to the cores of super-Earths bolsters the chance that they would host a strong, long-lived dynamo.

The Urey ratio is a fundamental parameter used to characterize the heat budget of the mantle. Following (*49*), we can define the conventional Urey ratio as Ur = (*H*_L_ + *H*_M_)/*Q*_M_ and the convective Urey ratio as Ur_conv_ = *H*_M_/*Q*_M_. With either definition, Urey ratios near or above ~0.5 to 1 would imply that internal heating powers mantle convection, as typically assumed for super-Earths (*8*, *50*, *90*). We found that the siderophile behavior of HPEs may instead lead to small Urey ratios for larger terrestrial planets, meaning that internal heating hardly contributes to the mantle’s heat budget. Rather, our models predict that heating from below and cooling from above drives mantle convection in super-Earths.

#### Initial conditions

Table S7 lists temperatures that are probably relevant to the evolution of planetary cores. Here, we assume that the silicate mantles and metallic cores have Earth-like abundances of iron and other major elements. Following previous studies, we use a fiducial “light element” to parameterize the composition of the core (*45*, *85*). Light elements lower the melting temperature of the core relative to that of a pure iron/nickel alloy. As the core cools, we assume that an inner core freezes outward from the center of the core. Blaske and O’Rourke (*45*) reported the temperature at the CMB when the inner core first nucleates (*T*_C_[*R_I_* = 0]). Noack and Lasbleis (*44*) calculated three other temperatures that are important to the core. First, the lowest temperature expected at the CMB is *T*_C,cold_, the adiabatic temperature at the bottom of the mantle, assuming that the mantle is ~2000 K at a depth of ~200 km. We use the same qualitative definition of *T*_C,cold_ in this study. However, we calculate the mantle adiabat using the procedure described above, because Noack and Lasbleis (*44*) used an approach that is valid only up to planetary masses of ~2 *M*_E_. Second, the hottest temperature that we assume for the top of the core is *T*_C,hot_, the liquidus temperature at the base of the mantle. We calculated this temperature from (*37*), assuming an Earth-like mantle composition. Last, an intermediate expected temperature is *T*_C,warm_, the solidus temperature of the basal mantle (fig. S11B). We also calculated this temperature following (*37*). We found that a core that is vigorously convective with a CMB temperature of *T*_C,hot_ or *T*_C,warm_ should be fully liquid. Because *T*_C,hot_ > *T*_C,warm_ > *T*_C,cold_, our assumption that the mantle remains fully solid is self-consistent as long as the core starts no hotter than *T*_C,warm_ and cools over time, which is how our models behave.

We started each model with the same thermal state to isolate and identify the effects of planetary mass and the partitioning behavior of the HPEs. The pressure and temperature conditions corresponding to core formation may vary depending on planet accretion processes (e.g., the number, timing, and energy of giant impacts). Nevertheless, at high pressures (>300 to 400 GPa) U and Th become strongly siderophile, and the partition coefficient of K only varies within a small range (Fig. 1). Thus, using partition coefficients of the HPEs obtained at somewhat different pressure and temperature conditions relevant to core formation for these large exoplanets does not influence our overall conclusions. Here, *t* = 0 represents the time at which the mantle has fully solidified, after it was at least partially molten in the aftermath of planetary accretion. At that time, we set the potential temperature of the mantle to *T*_M_ = 2000 K. We assume that the lithosphere has an initial thickness of *d_u_* = 100 km but contains no HPEs to start. We set *T*_C_ = *T*_C,warm_ although hotter temperatures are also plausible, which would lead to stronger and longer-lived dynamos and melt production. We used Euler’s method to solve the governing equations with a timestep of Δ*t* = 0.5 thousand years until *t* = 20 Gyr, at which time melt production and the dynamo had ceased in all models. We verified that using a smaller timestep would not change the model output.

#### Sensitivity tests

To present the results presented in Fig. 3, we ran 72 models. We explored all combinations of four planetary masses, two partitioning behaviors for HPEs (i.e., siderophile versus lithophile), three viscosity models for post-perovskite in the lower mantle, and three values of the thermal conductivity of the core (i.e., 75, 100, and 125% of the nominal value determined as described above). Figures S12 to S17 show the detailed output from the models for a 4-*M*_E_ super-Earth using the nominal thermal conductivity for the core. The last nonzero values in subpanels (F) and (I) were extracted for Fig. 3. All the models display similar trends. The planets start hot and cool monotonically over time. The heat fluxes out of the mantle and core are relatively huge at initially but gradually decrease as the mantle becomes increasingly viscous. The lithosphere quickly adjusts from its initial thickness to an equilibrium value and then steadily thickens as the mantle cools. Melt production slows over time because the initial and final depths of melting gradually converge. Last, the predicted magnetic field strength mostly declines over billions of years. Our models predict a spike in field strength lasting <1 Gyr when the inner core nucleates. However, more detailed studies of Earth’s dynamo indicate that the nucleation of the inner core might not produce an observable change in the surface field intensity (*91*, *92*), which agrees with the lack of scientific consensus about whether any such spike exists in Earth’s paleomagnetic record. The partitioning behavior of HPEs does not affect these trends. However, moving HPEs to the core keeps internal temperatures hot and heat fluxes high in these models, leading to longer predicted lifetimes of near-surface melt production and magnetic fields.

Figures S18 and S19 show the summary statistics from the models with higher- and lower-than-nominal thermal conductivity in the core, respectively. Relative to Fig. 3, these plots show clear trends. The thermal conductivity of the core does not affect the lifetime of melt production near the surface, which instead depends on the mantle viscosity and the partitioning behavior of the HPEs. However, higher thermal conductivity in the core always leads to shorter-lived and weaker dynamo. Raising or lowering the thermal conductivity of the core by ~25% can reduce or lengthen the predicted dynamo lifetime by several billion years, respectively. The predicted magnetic fields at the surface can likewise change by ~10 mT because thermal conduction acts as a dissipation sink in the core (*85*). For any value of the thermal conductivity of the core, the siderophile behavior of HPEs makes larger super-Earths relatively more likely to sustain global magnetic fields.

### Caveats and priorities for future studies

We designed our thermal evolution models to illustrate the first-order effects of siderophile HPEs on the evolution of super-Earth exoplanets. This approach, following many previous studies, necessarily embraced several limitations, including but not limited to the following:

1) Our models were parametrized and one-dimensional. Mantle convection is intrinsically three-dimensional (3D), featuring latitudinal and longitudinal variations in temperature, composition, and other properties. The scaling laws for stagnant-lid convection have been benchmarked to 3D models, but 3D models could still capture important aspects of planetary evolution that are not illustrated here (*50*).

2) Our models assumed a constant surface temperature and thus only one mode of mantle convection. Stagnant-lid convection has been considered the most natural mode of mantle convection for planets with surfaces that are too hot for stable liquid water. However, the nearest such planet (Venus) may operate in a different mode of mantle convection facilitated by intrusive magmatism (*93*). Real planets also have atmospheres that evolve in response to stellar evolution and mantle degassing. Climatic shifts can cause the regime of mantle convection to change, plausibly more than once during a planet’s lifetime (*94*, *95*). Fully coupled models of atmosphere-surface-mantle-core evolution would be critical to studies that attempt to reproduce the histories of individual planets.

3) Studying habitable worlds would require more complicated models, which would describe the atmosphere-surface-interior cycling of key elements such as hydrogen, water, carbon, and sulfur in detail (*90*, *96*). Habitable planets may feature a plate tectonic-like regime of mantle convection, including analogues to both the oceanic and continental crust. Modeling the influence of a planetary magnetic field on surface habitability is also complex and likely varies based on the type of parent star and the planetary orbit (*97*–*99*).

4) Our models do not include a BMO. Most studies assume that planetary mantles solidify from the bottom up. However, the mantles of Earth-sized and larger planets may crystallize from the center outward. The surficial magma ocean would solidify within ~10 to 100 Myr, depending on the properties of any overlying atmosphere (*73*). The BMO would remain molten for billions of years (*74*) because the overlying solid mantle acts as a thermal blanket, and the neighboring core serves as a heat sink. Notably, molten silicates become electrically conductive at extreme pressures and temperatures, meaning that BMOs in super-Earths could host dynamos (*62*), as proposed for Earth’s ancient BMO (*100*, *101*). One would expect substantial amounts of chemical exchange, including of HPEs, between the core and the BMO across the liquid-liquid interface (*102*).

These limitations are opportunities for future research. Regardless of the model used, the partitioning behavior of HPEs may markedly influence the thermal evolution of terrestrial exoplanets. For example, many previous efforts have ignored the cooling of the core entirely, assuming instead that super-Earths have mantles that are only internally heated (*82*, *90*). Our results imply that this foundational assumption is incorrect for larger planets and that a new foundation should be built for sophisticated models of terrestrial exoplanets, including potentially habitable super-Earths.
