# Supplementary material for: Radiogenic heating sustains long-lived volcanism and magnetic dynamos in super-Earths
Source: Sci Adv. 2024 Sep 13;10(37):eado7603. doi: 10.1126/sciadv.ado7603 (PMC11397497; doi:10.1126/sciadv.ado7603)
Supplement: Supplementary file 1 — Figs. S1 to S19 Tables S1 to S7 [file sciadv.ado7603_sm.pdf]

Science Advances
AAAS

Supplementary Materials for

Radiogenic heating sustains long-lived volcanism and magnetic dynamos in super-Earths

Haiyang Luo *et al.*

Corresponding author: Haiyang Luo, haiyang.luo@princeton.edu; Jie Deng, jie.deng@princeton.edu

Sci. Adv. **10**, eado7603 (2024)
DOI: 10.1126/sciadv.ado7603

This PDF file includes:

Figs. S1 to S19
Tables S1 to S7

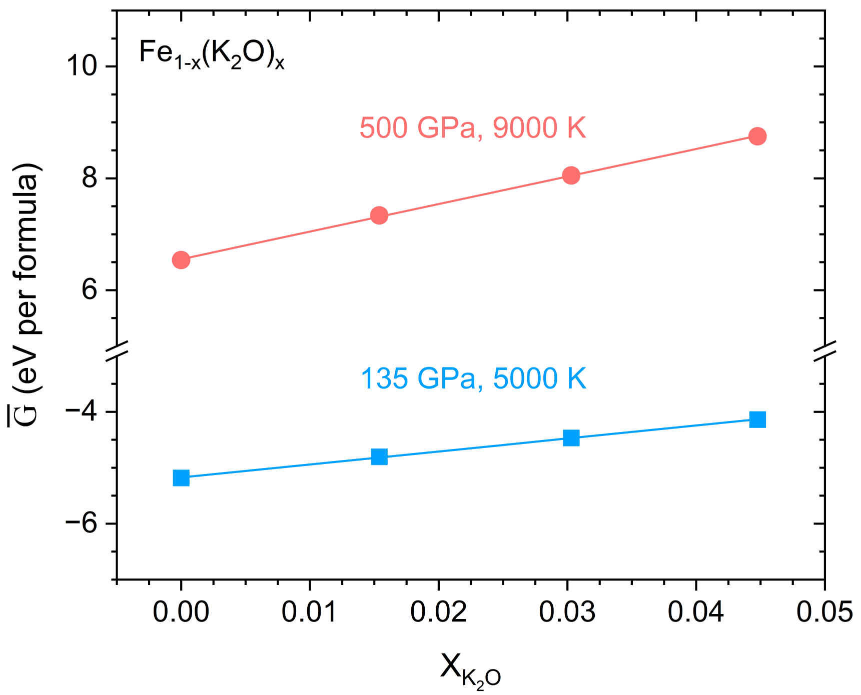

Fig. S1. Calculated Gibbs free energies of $Fe_{1-x}(K_2O)_x$ melt as a function of K_2O mole fraction (X_{K_2O}).

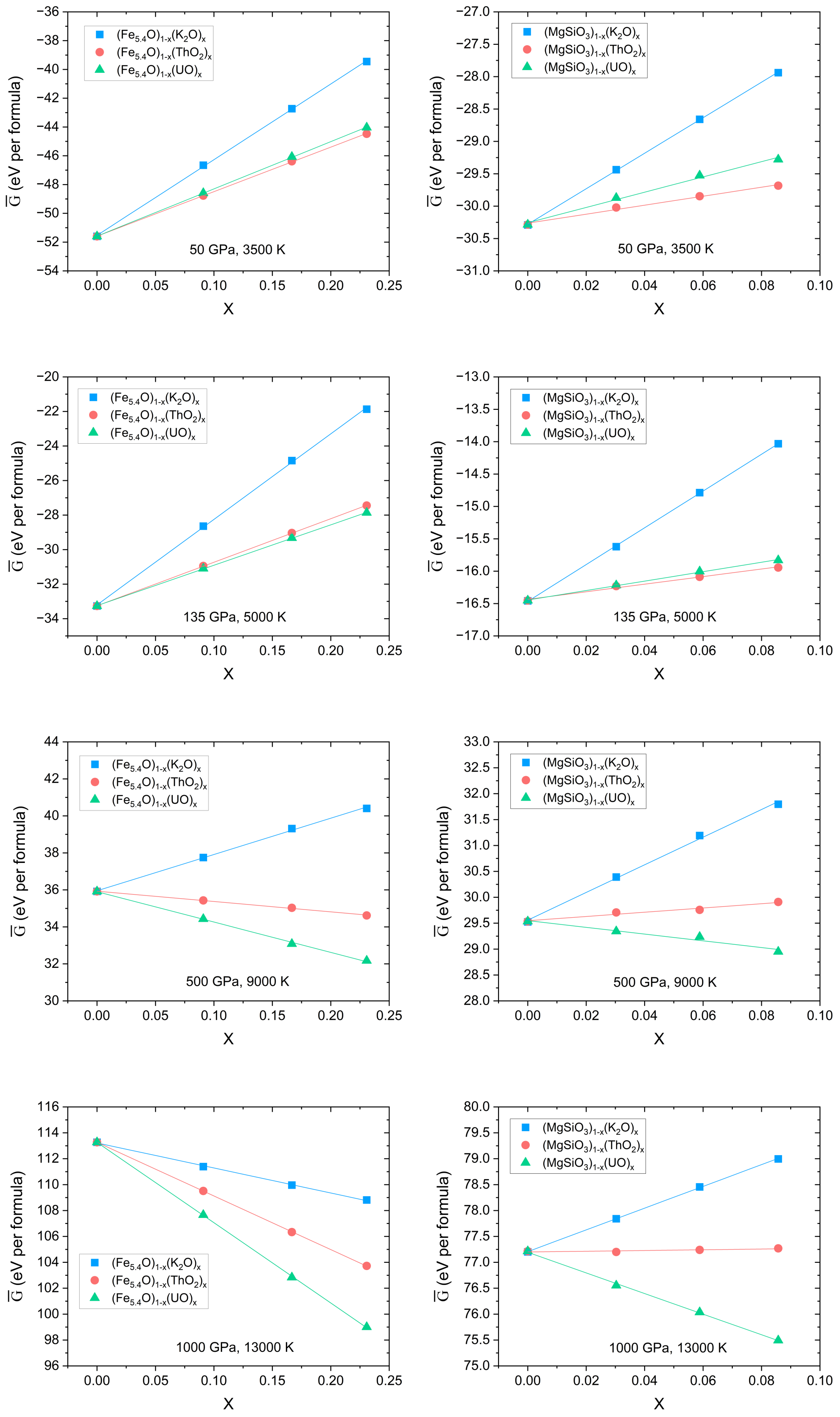

Fig. S2. Gibbs free energies variation with respect to HPEs concentrations. Calculated Gibbs free energies of $(Fe_{5.4}O)_{1-x}(K_2O)_x$, $(MgSiO_3)_{1-x}(K_2O)_x$, $(Fe_{5.4}O)_{1-x}(ThO_2)_x$, $(MgSiO_3)_{1-x}(ThO_2)_x$, $(Fe_{5.4}O)_{1-x}(UO)_x$, and $(MgSiO_3)_{1-x}(UO)_x$ melts as a function of K_2O, ThO_2, and UO mole fractions, respectively.

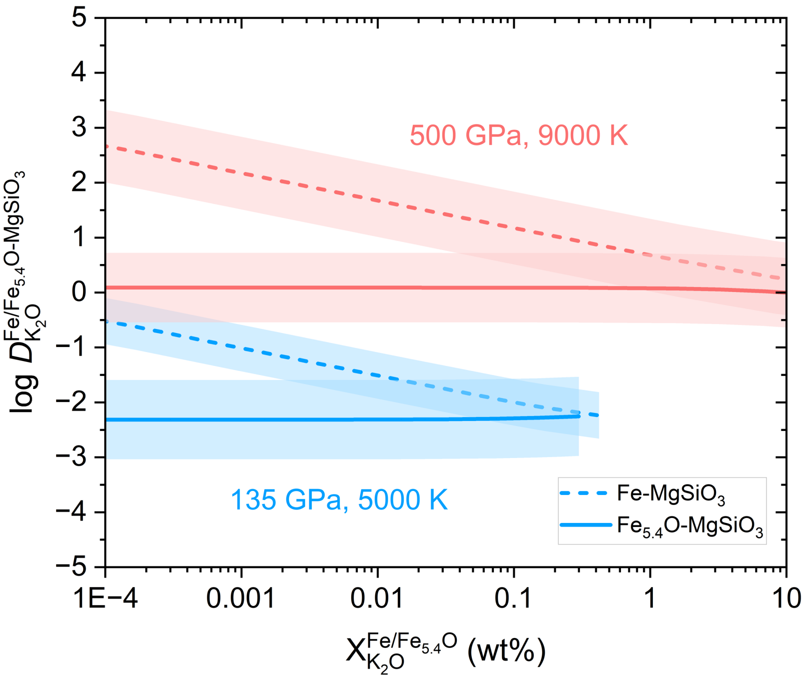

Fig. S3. Comparison of the partition coefficients (wt%) of K_2O in Fe-MgSiO$_3$ and Fe$_{5.4}$O-MgSiO$_3$ systems at 135 and 500 GPa. The shaded regions represent the errors. The divergence at low K_2O concentration is due to the difference in mixing entropy.

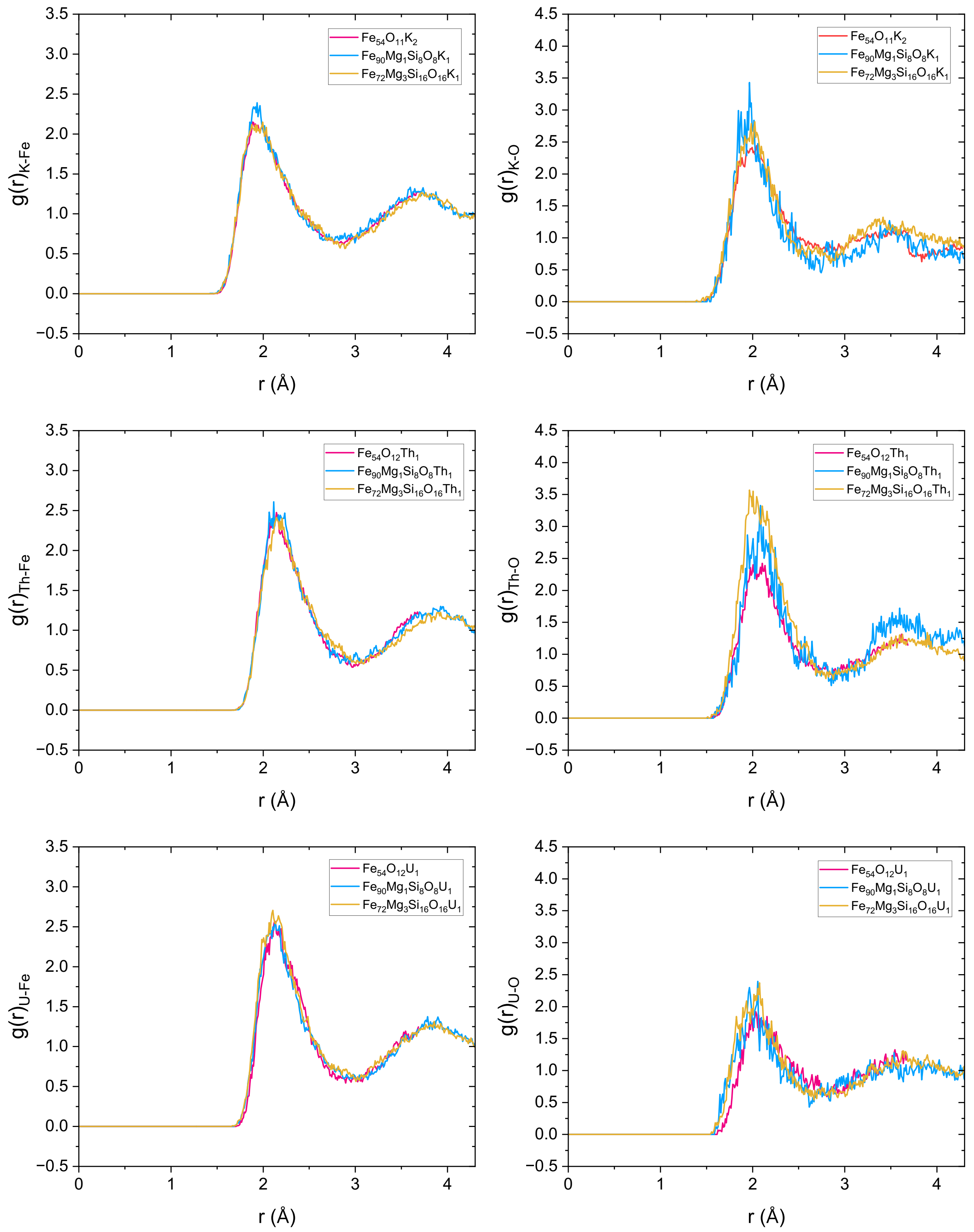

Fig. S4. Calculated K/Th/U-Fe/O radial distribution functions in liquid metal of different compositions at 500 GPa and 9000 K. Varying bulk compositions does not significantly change the characteristics of the local structure of K/Th/U with respect to Fe/O in liquid metal.

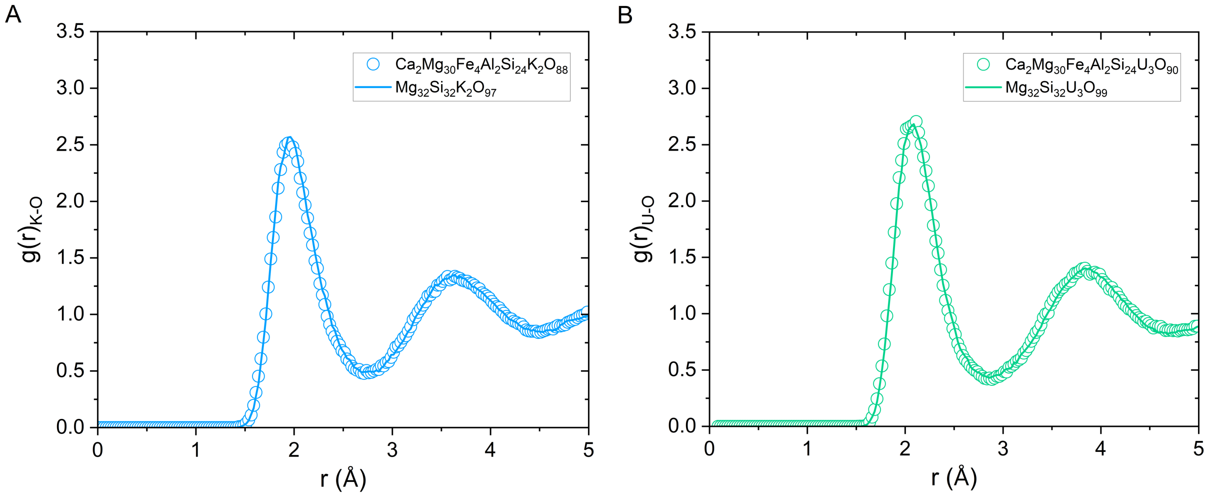

Fig. S5. Calculated K-O (A) and U-O (B) radial distribution functions in silicate melt at 500 GPa and 9000 K. Varying bulk compositions does not significantly change the characteristics of the local structure of K/U with respect to O in silicate melt.

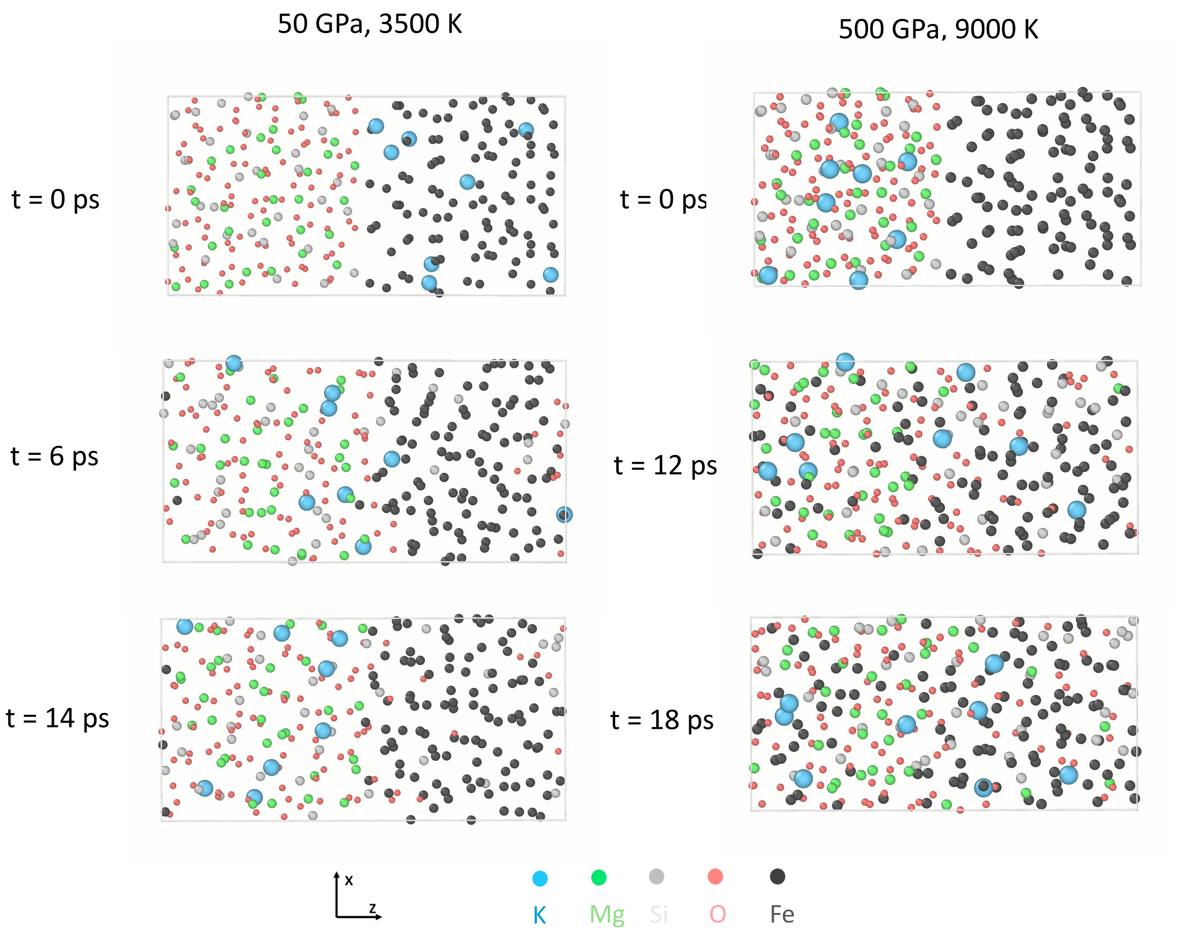

Fig. S6. Two-phase molecular dynamics simulations of potassium partitioning between metal and silicate melts at 50 GPa/3500 K and 500 GPa/9000 K. The initial configurations and the snapshots at different picoseconds (ps) are shown. More Mg, Si, and O atoms dissolve into the liquid metal at 500 GPa compared to 50 GPa. Potassium shows highly lithophile behavior at 50 GPa, but more or less equally partitioning between metal and silicate melts at 500 GPa, consistent with our results based on thermodynamic integration.

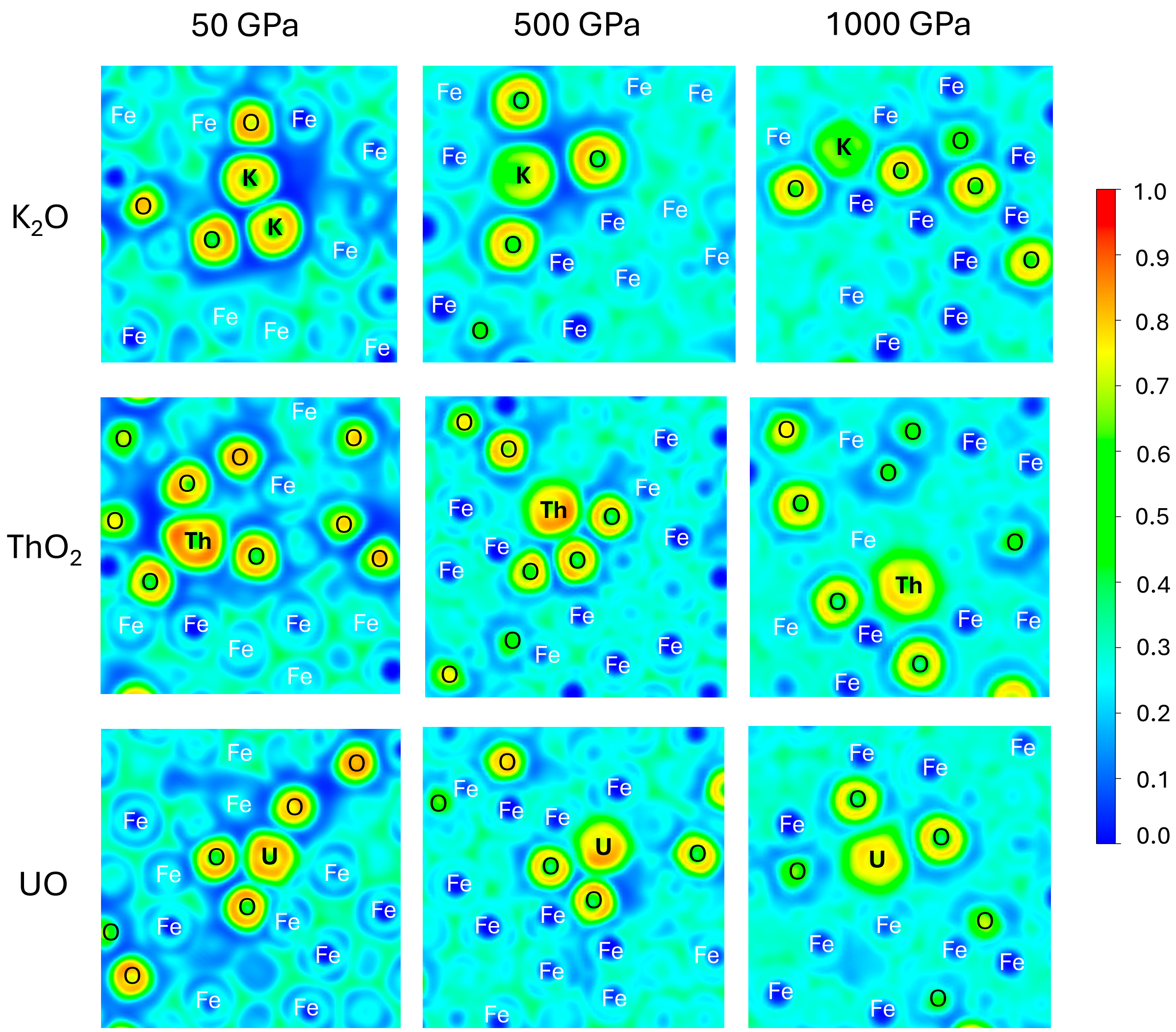

Fig. S7. Electron localization function (ELF) maps in a plane through the $(Fe_{54}O_{10})(K_2O)_1$, $(Fe_{54}O_{10})(ThO_2)_1$, and $(Fe_{54}O_{10})(UO)_1$ cells. The ELF shown here are at 50, 500, and 1000 GPa, corresponding to 3500, 9000, and 13000 K, respectively. ELF values ranging from 0.0 to 1.0 represent a continuum between complete electron delocalization and complete electron localization.

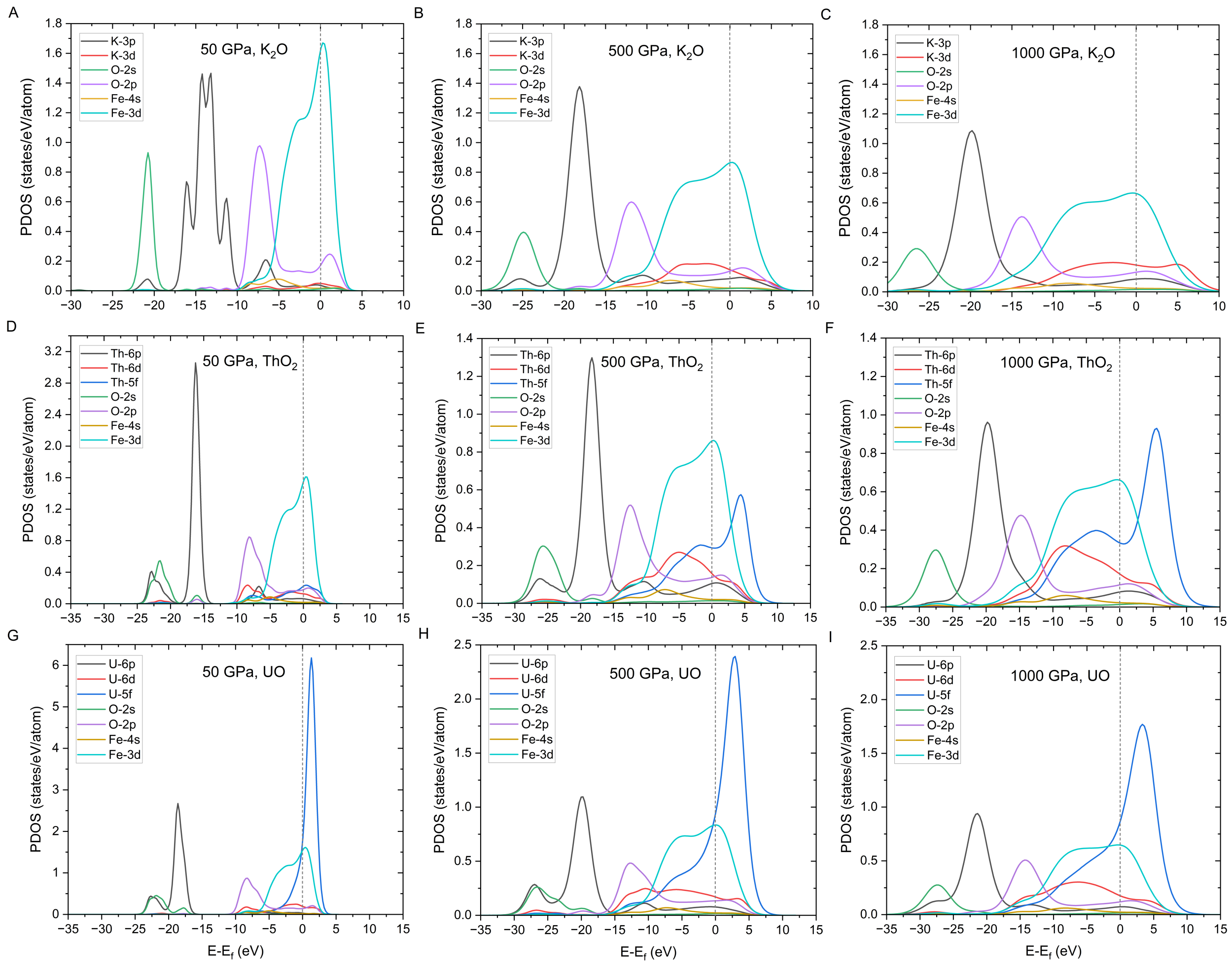

Fig. S8. Projected densities of states (PDOS) of $(Fe_{54}O_{10})(K_2O)_1$, $(Fe_{54}O_{10})(ThO_2)_1$, and $(Fe_{54}O_{10})(UO)_1$. The PDOS shown here **(A-I)** are at 50, 500, and 1000 GPa, corresponding to 3500, 9000, and 13000 K, respectively.

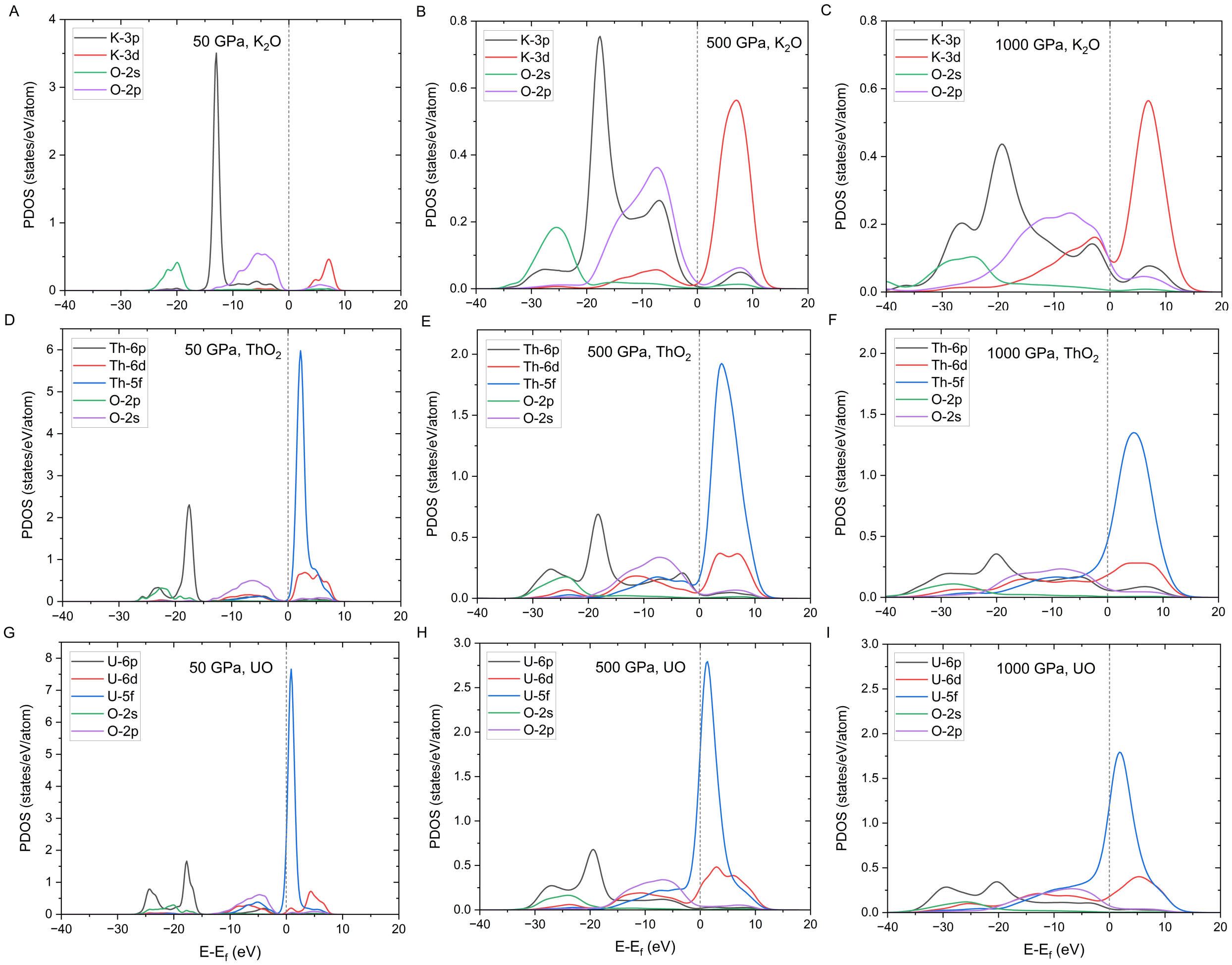

Fig. S9. Projected densities of states (PDOS) of $(MgSiO_3)_{32}(K_2O)_1$, $(MgSiO_3)_{32}(ThO_2)_1$, and $(MgSiO_3)_{32}(UO)_1$. The PDOS shown here **(A-I)** are at 50, 500, and 1000 GPa, corresponding to 3500, 9000, and 13000 K, respectively.

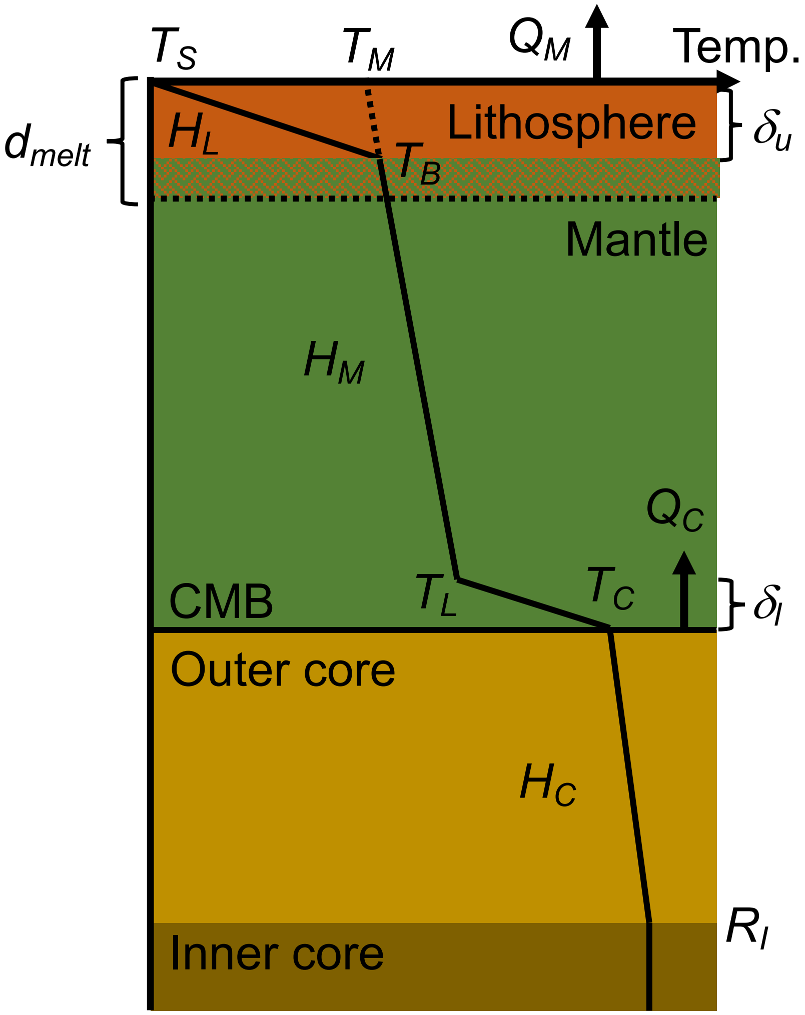

Fig. S10. Thermal structure of a rocky planet in our simple, parameterized models. Our models tracked the mantle potential temperature (T_M) and the temperature at the surface (T_S), at the base of the lithosphere (T_B), in the lower mantle (T_L) and at the top of the core (T_C). We calculated the radiogenic heat production in the lithosphere (H_L), mantle (H_M) and core (H_C) over time, as well as the heat flux out of the mantle (Q_M) and across the core-mantle boundary (CMB) (Q_C). The thicknesses of the thermal boundary layers at the top and bottom of the mantle are d_u and d_l, respectively. Following our previous study (*45*) and references therein, we calculated when an inner core would nucleate and how its radius (R_I) would increase over time. Near the surface, the amount of melt production depends on the depth at which melting begins below the lithosphere (d_{melt}).

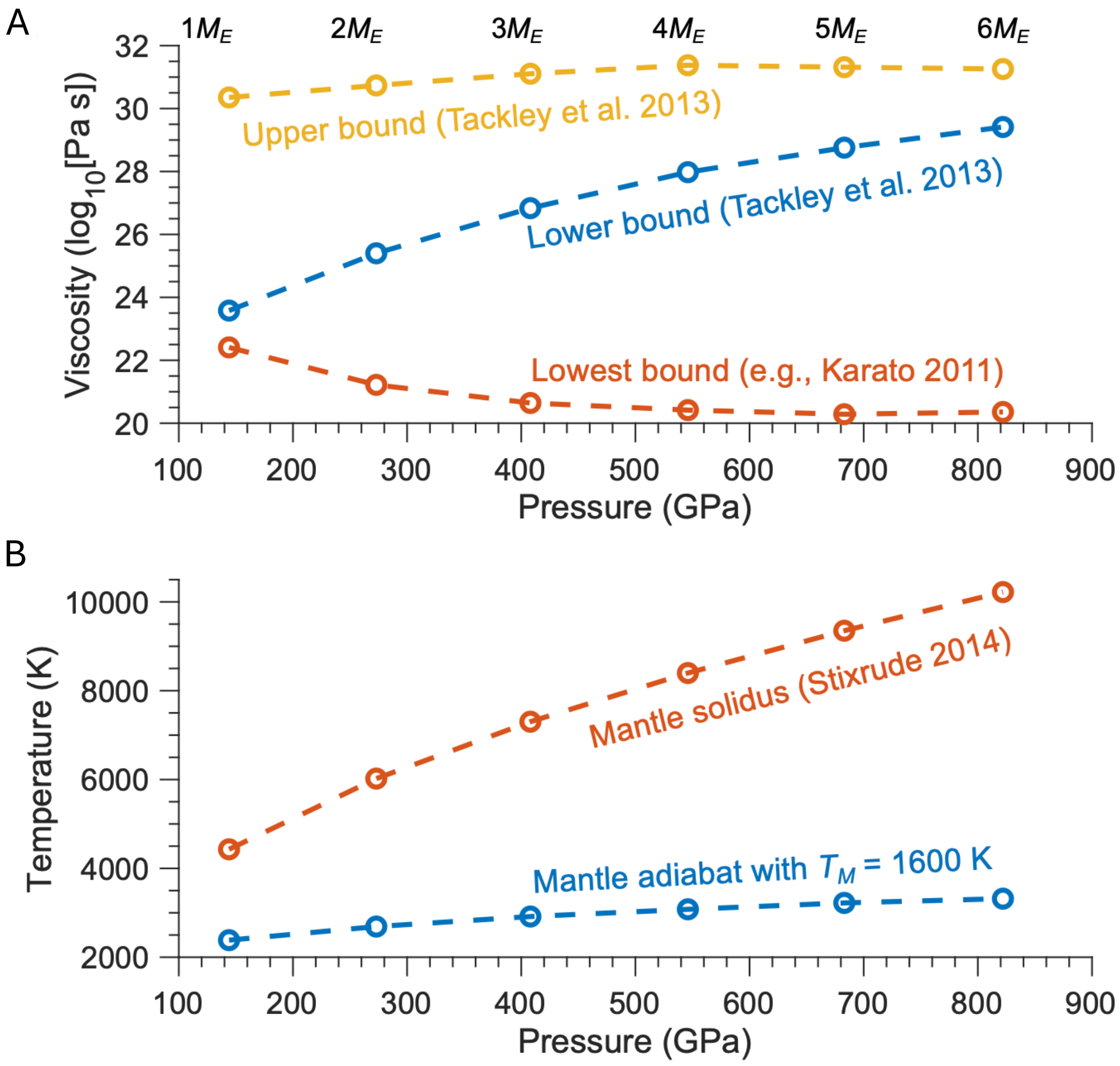

Fig. S11. Viscosity and temperature in the lower mantle. **(A)** Viscosity in the lower mantle according to the lower (blue) and upper (yellow) bounds from (*51*) and a "lowest bound" (red) based on (*52*). Circles show the pressure at the core-mantle boundary for super-Earths of 1–6 Earth-masses. **(B)** Comparison of the solidus (red) and adiabatic (blue) temperature in the lower mantle, with the mantle potential temperature set to 1600 K. These temperatures were used to calculate the viscosities plotted above. Our models start with a mantle potential temperature of 2000 K and a CMB temperature equal to the mantle solidus. The models then self-consistently predict that the mantle remains fully solid. Future studies should explore hotter starts and a basal magma ocean.

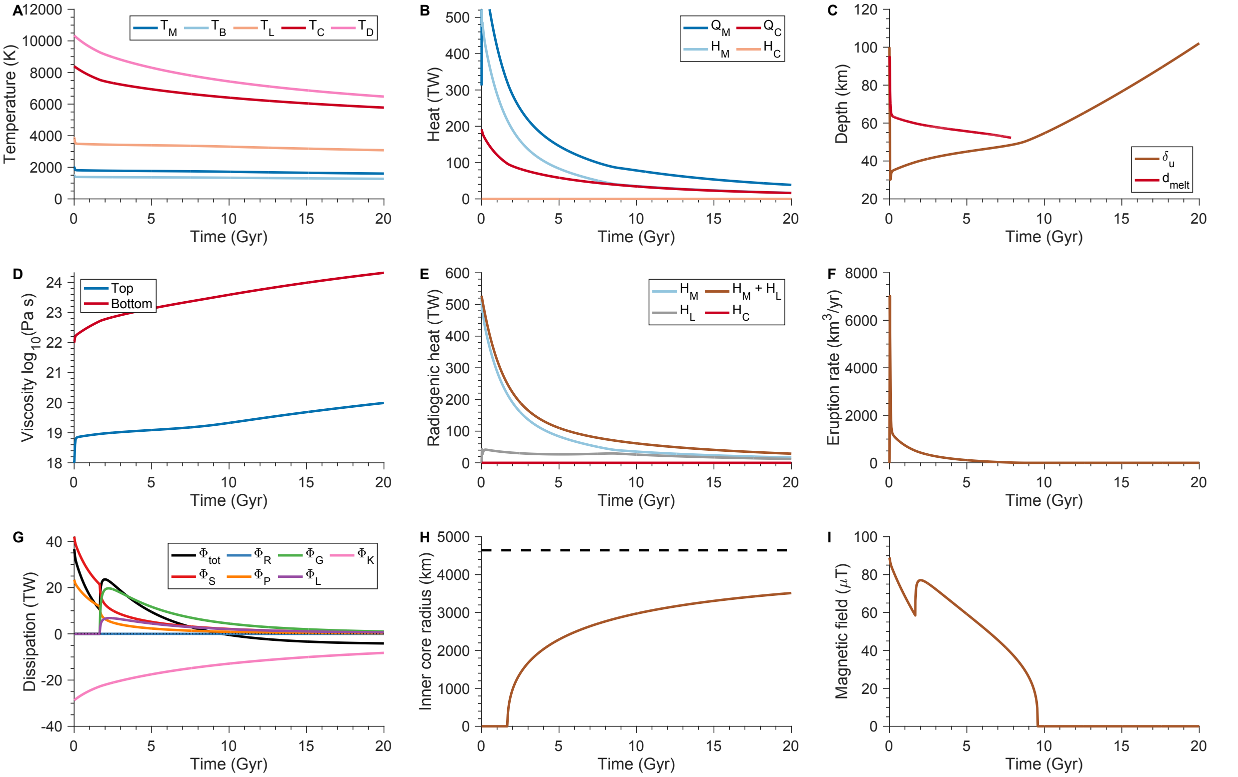

Fig. S12. Thermal evolution model for a 4-M_E planet with no HPEs in the core, using the lower-bound model for the viscosity of post-perovskite. Output includes (**A**) temperatures shown in Fig. S3 plus the average temperature in the liquid part of the core (T_D); (**B**) heat fluxes and radiogenic heat production; (**C**) depths to the base of the lithosphere and where partial melting starts; (**D**) viscosities of the upper and lower thermal boundary layers in the mantle; (**E**) the complete budget of radiogenic heating; (**F**) the rate of melt production, without distinction between extrusive volcanism and intrusive magmatism; (**G**) the dissipation budget for the core (*45*); (**H**) the radius of the inner core; and (**I**) the predicted strength of the magnetic field on the surface at the equator, according to mixing length theory.

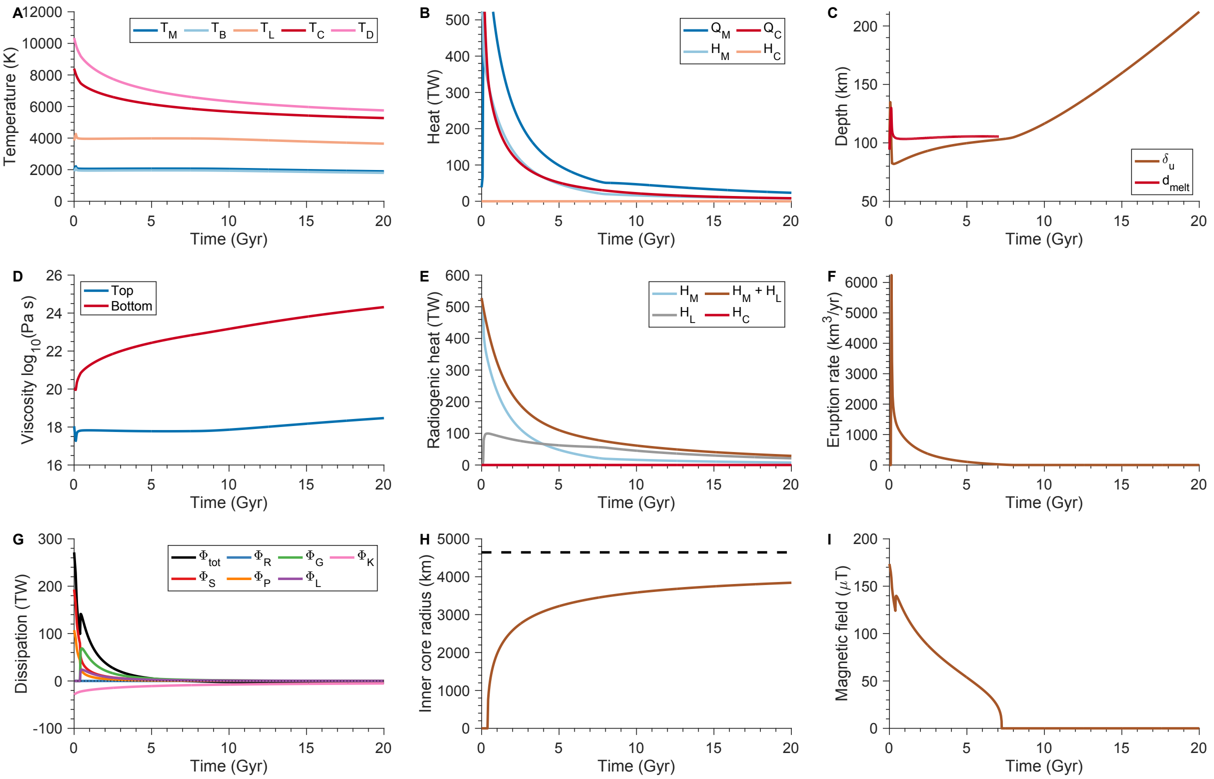

Fig. S13. Thermal evolution model for a 4-M_E planet with no HPEs in the core, using the upper-bound model for the viscosity of post-perovskite. Subplots are the same as in Fig. S12.

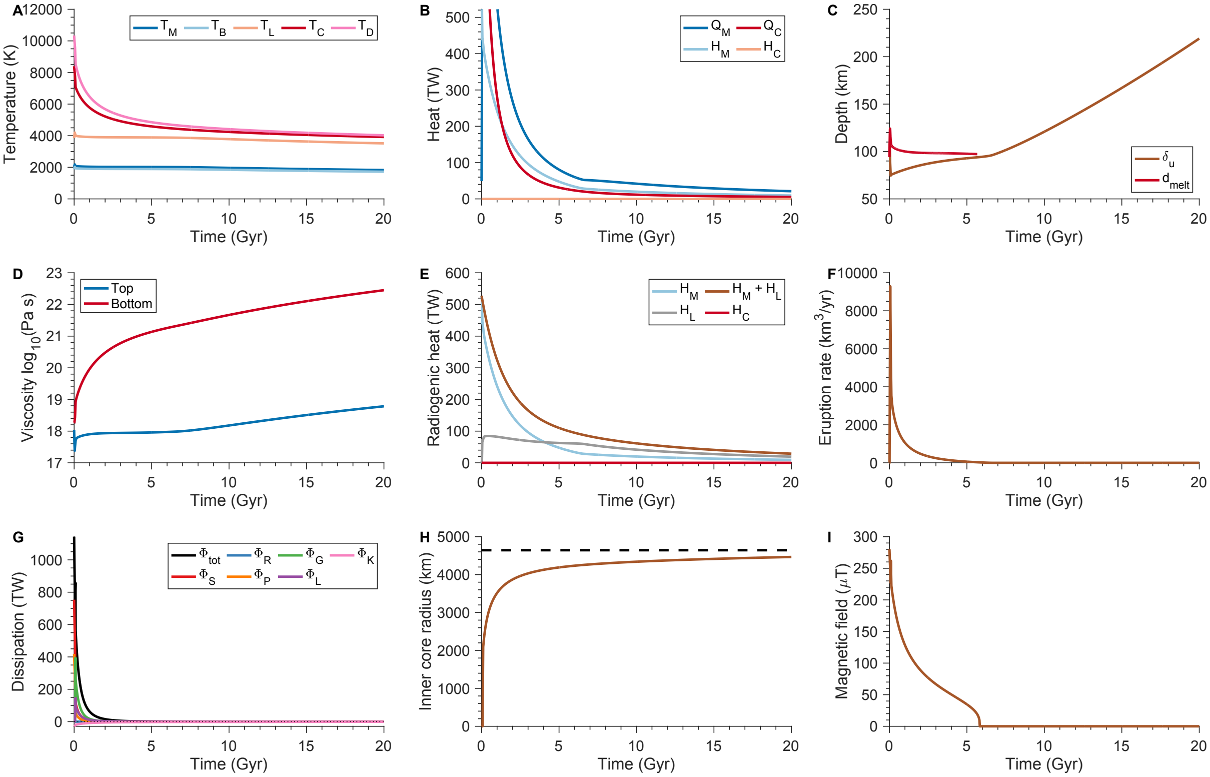

Fig. S14. Thermal evolution model for a 4-M_E planet with no HPEs in the core, using the lowest-bound model for the viscosity of post-perovskite. Subpanels are the same as in Fig. S12.

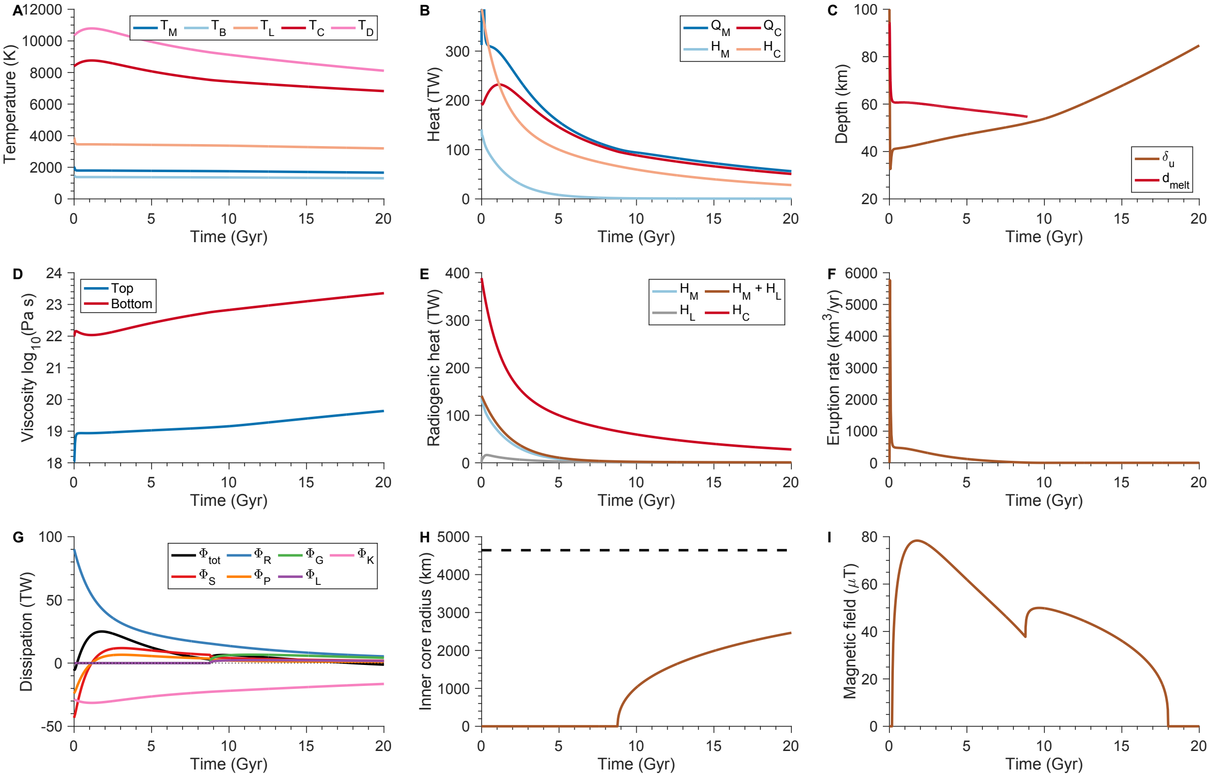

Fig. S15. Thermal evolution model for a 4-*M_E* planet with HPEs partitioned assuming metal-silicate equilibration at 500 GPa and 9000 K, using the lower-bound model for the viscosity of post-perovskite. Subpanels are the same as in Fig. S12.

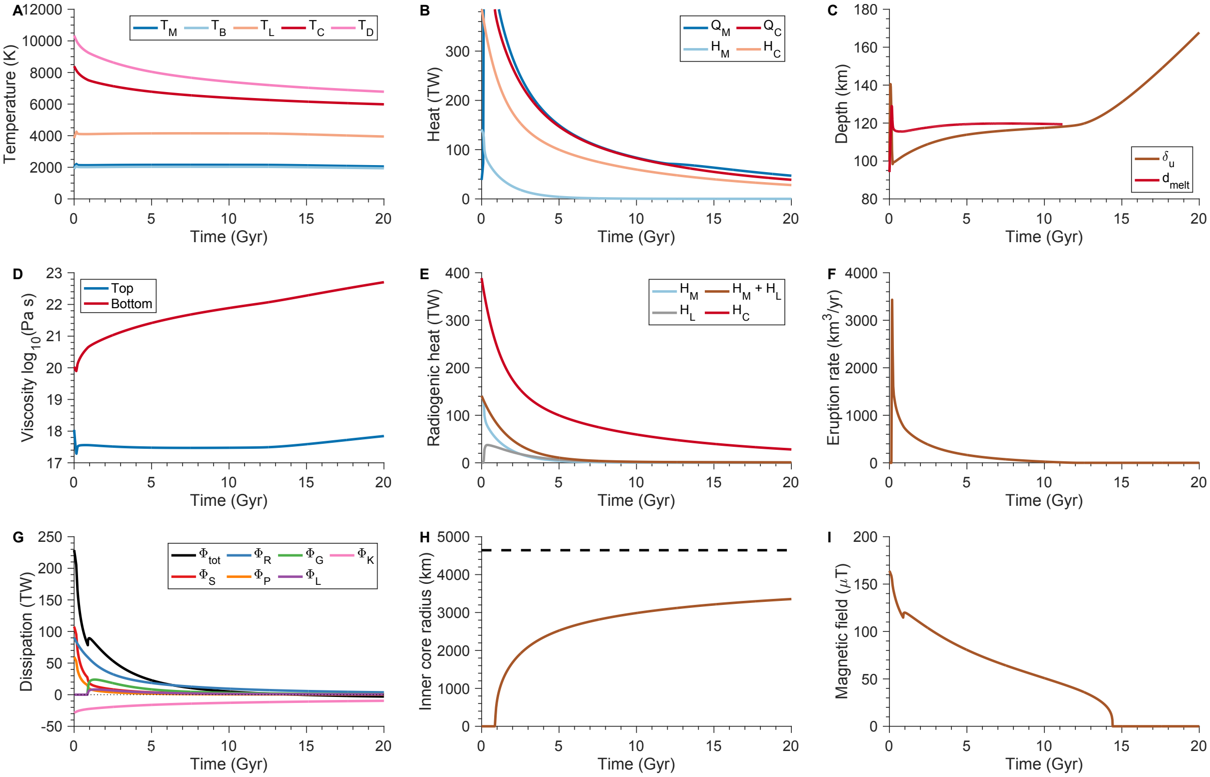

Fig. S16. Thermal evolution model for a 4-M_E planet with HPEs partitioned assuming metal-silicate equilibration at 500 GPa and 9000 K, using the upper-bound model for the viscosity of post-perovskite. Subpanels are the same as in Fig. S12.

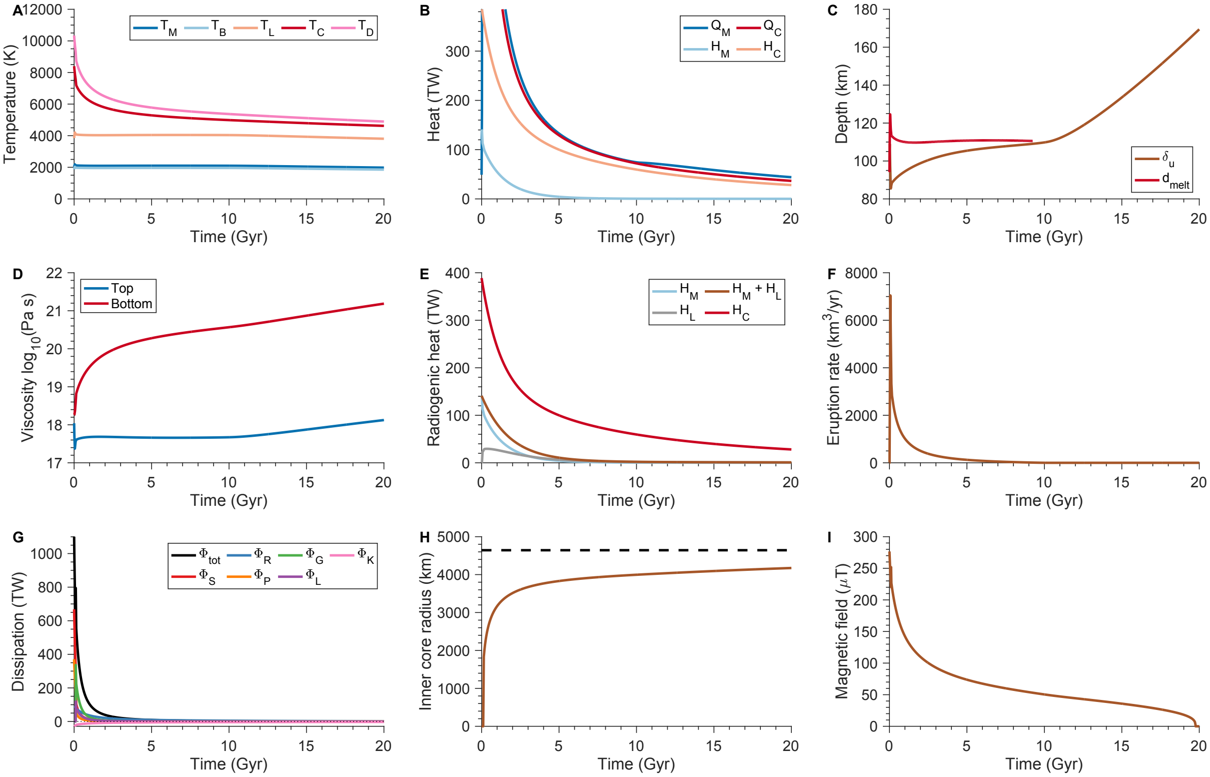

Fig. S17. Thermal evolution model for a 4-M_E planet with HPEs partitioned assuming metal-silicate equilibration at 500 GPa and 9000 K, using the lowest-bound model for the viscosity of post-perovskite. Subpanels are the same as in Fig. S12.

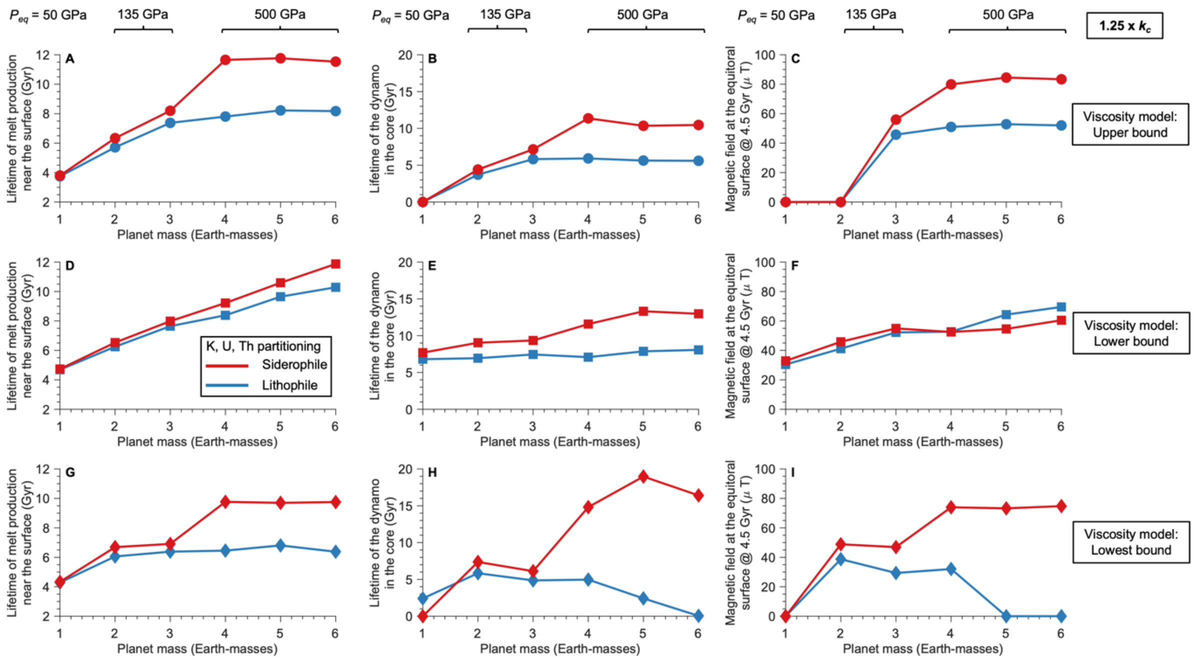

Fig. S18. Same as Fig. 3, but with the thermal conductivity of the core increased by 25% relative to its nominal value in all models.

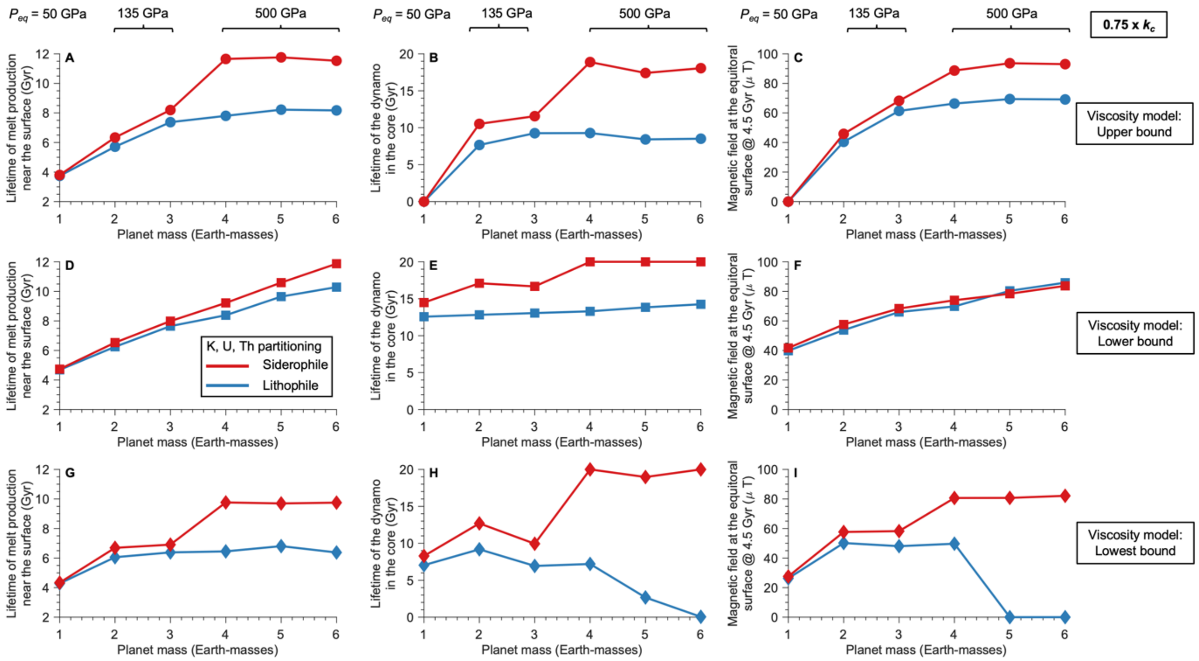

Fig. S19. Same as Fig. 3, but with the thermal conductivity of the core reduced by 25% relative to its nominal value in all models.

Table S1. Gibbs free energies. Calculated Gibbs free energies of iron/iron-alloy and silicate melts with difference concentrations of K_2O, ThO_2, and UO at 50, 135, 500, and 1000 GPa, corresponding to 3500, 5000, 9000, and 13000 K, respectively.

P (GPa)	T (K)	System	V (Å^3/atom)	$\bar{G}$ (eV/atom)	System	V (Å^3/atom)	$\bar{G}$ (eV/atom)
50	3500	$Fe_{54}O_{10}$	9.227	–8.063±0.004	$(MgSiO_3)_{32}$	8.072	–6.058±0.003
135	5000		7.972	–5.198±0.005		6.625	–3.292±0.004
500	9000		6.008	5.612±0.010		4.695	5.905±0.007
1000	13000		4.993	17.699±0.016		3.780	15.440±0.008
50	3500	$(Fe_{54}O_{10})(K_2O)_1$	9.469	–7.660±0.005	$(MgSiO_3)_{32}(K_2O)_1$	8.149	–5.960±0.004
135	5000		8.061	–4.703±0.005		6.697	–3.163±0.004
500	9000		6.015	6.198±0.009		4.730	6.153±0.005
1000	13000		4.988	18.288±0.011		3.811	15.759±0.006
50	3500	$(Fe_{54}O_{10})(K_2O)_2$	9.628	–7.325±0.005	$(MgSiO_3)_{32}(K_2O)_2$	8.261	–5.870±0.003
135	5000		8.151	–4.259±0.008		6.752	–3.029±0.004
500	9000		6.029	6.740±0.010		4.768	6.389±0.006
1000	13000		4.986	18.851±0.015		3.838	16.069±0.007
50	3500	$(Fe_{54}O_{10})(K_2O)_3$	9.770	–7.026±0.005	$(MgSiO_3)_{32}(K_2O)_3$	8.377	–5.786±0.004
135	5000		8.233	–3.894±0.006		6.825	–2.906±0.004
500	9000		6.036	7.195±0.008		4.803	6.585±0.006
1000	13000		4.986	19.378±0.012		3.862	16.360±0.007
50	3500	$(Fe_{54}O_{10})(ThO_2)_1$	9.348	–8.007±0.004	$(MgSiO_3)_{32}(ThO_2)_1$	8.074	–6.078±0.003
135	5000		8.039	–5.081±0.005		6.625	–3.286±0.004
500	9000		6.027	5.817±0.009		4.737	6.014±0.005
1000	13000		5.006	17.980±0.012		3.817	15.629±0.006
50	3500	$(Fe_{54}O_{10})(ThO_2)_2$	9.469	–7.952±0.004	$(MgSiO_3)_{32}(ThO_2)_2$	8.151	–6.113±0.004
135	5000		8.100	–4.978±0.006		6.698	–3.295±0.004
500	9000		6.047	6.005±0.011		4.522	6.095±0.005
1000	13000		5.018	18.228±0.012		3.846	15.820±0.007
50	3500	$(Fe_{54}O_{10})(ThO_2)_3$	9.552	–7.921±0.005	$(MgSiO_3)_{32}(ThO_2)_3$	8.247	–6.148±0.003
135	5000		8.161	–4.888±0.007		6.788	–3.302±0.004
500	9000		6.069	6.165±0.009		4.772	6.194±0.006
1000	13000		5.031	18.472±0.011		3.879	16.003±0.007
50	3500	$(Fe_{54}O_{10})(UO)_1$	9.329	–8.099±0.005	$(MgSiO_3)_{32}(UO)_1$	8.134	–6.085±0.003
135	5000		8.040	–5.183±0.005		6.679	–3.304±0.004
500	9000		6.049	5.738±0.009		4.814	5.977±0.006
1000	13000		5.026	17.943±0.011		3.816	15.594±0.007
50	3500	$(Fe_{54}O_{10})(UO)_2$	9.427	–8.131±0.004	$(MgSiO_3)_{32}(UO)_2$	8.216	–6.121±0.003
135	5000		8.113	–5.175±0.005		6.734	–3.318±0.004
500	9000		6.087	5.838±0.008		4.775	6.061±0.005
1000	13000		5.057	18.147±0.011		3.848	15.764±0.007
50	3500	$(Fe_{54}O_{10})(UO)_3$	9.511	–8.178±0.005	$(MgSiO_3)_{32}(UO)_3$	8.295	–6.173±0.003
135	5000		8.180	–5.175±0.005		6.775	–3.338±0.004
500	9000		6.127	5.975±0.009		4.814	6.104±0.006
1000	13000		5.088	18.386±0.010		3.880	15.917±0.007
135	5000	Fe_{64}	8.469	–5.182±0.007			
500	9000		6.434	6.538±0.012			
135	5000	$(Fe_{64})(K_2O)_1$	8.539	–4.660±0.006			
500	9000		6.415	7.116±0.010			
135	5000	$(Fe_{64})(K_2O)_2$	8.600	–4.212±0.006			
500	9000		6.405	7.589±0.011			
135	5000	$(Fe_{64})(K_2O)_3$	8.658	–3.800±0.005			
500	9000		6.396	8.032±0.010			

Table S2. Chemical potentials and partition coefficients. Calculated chemical potentials and partition coefficients of K_2O, ThO, ThO_2, UO and UO_2 between metal ($Fe_{5.4}O$) and silicate ($MgSiO_3$) melts. Partition coefficients are calculated using mole fractions.

P (GPa)	*T* (K)	System (*i*)	$\bar{\mu}_i^{\mathrm{Fe_{5.4}O}}$ (eV)	$\bar{\mu}_i^{\mathrm{MgSiO_3}}$ (eV)	$\log_{10}D$
50	3500	K_2O	1.090±0.558	–2.842±0.224	–2.937±0.867
135	5000		16.332±0.681	11.938±0.218	–2.320±0.721
500	9000		55.571±0.521	56.252±1.001	0.085±0.632
1000	13000		93.896±0.459	98.211±0.197	0.731±0.194
50	3500	ThO	–13.282±0.216	–15.021±0.712	–2.318±1.072
135	5000		–2.487±0.175	–3.969±0.225	–1.307±0.287
500	9000		30.176±0.442	33.019±0.919	1.778±0.571
1000	13000		64.299±0.460	71.055±0.498	2.806±0.263
50	3500	ThO_2	–20.637±0.229	–23.320±0.463	–3.093±0.744
135	5000		–8.030±0.104	–10.581±0.377	–1.801±0.394
500	9000		30.350±0.179	33.655±0.566	2.621±0.333
1000	13000		71.840±0.156	77.915±0.297	3.126±0.130
50	3500	UO	–18.741±0.200	–18.462±0.708	0.588±1.060
135	5000		–9.835±0.167	–9.156±0.218	0.871±0.277
500	9000		19.496±0.441	23.050±0.918	2.177±0.570
1000	13000		51.234±0.459	57.294±0.495	2.536±0.262
50	3500	UO_2	–25.939±0.245	–26.794±0.469	–0.460±0.763
135	5000		–15.370±0.123	–15.871±0.382	0.266±0.405
500	9000		19.674±0.181	23.791±0.571	3.076±0.335
1000	13000		58.764±0.158	64.282±0.301	2.910±0.132

The calculated partition coefficients can be represented by the exponential equations:

$\log_{10} D_{\mathrm{K_2O}}^{\mathrm{metal-silicate}} = 0.927 - 4.723\exp(-P/311.7)$;

$\log_{10} D_{\mathrm{ThO}}^{\mathrm{metal-silicate}} = 3.126 - 6.655\exp(-P/326.6)$;

$\log_{10} D_{\mathrm{ThO_2}}^{\mathrm{metal-silicate}} = 3.490 - 8.233\exp(-P/261.4)$;

$\log_{10} D_{\mathrm{UO}}^{\mathrm{metal-silicate}} = 2.663 - 2.690\exp(-P/321.2)$;

$\log_{10} D_{\mathrm{UO_2}}^{\mathrm{metal-silicate}} = 2.966 - 5.333\exp(-P/168.1)$.

Table S3. Gibbs free energies of complex systems. Calculated Gibbs free energies of iron-alloy ($Fe_{54}Mg_1Si_3O_{10}$) and silicate melts ($Ca_2Mg_{30}Fe_4Al_2Si_{24}O_{87}$) with difference concentrations of K_2O and UO at 500 GPa and 9000 K.

P (GPa)	T (K)	System	V (Å^3/atom)	$\bar{G}$ (eV/atom)	System	V (Å^3/atom)	$\bar{G}$ (eV/atom)
500	9000	$Fe_{54}Mg_1Si_3O_{10}$	6.019	6.049±0.009	$Ca_2Mg_{30}Fe_4Al_2Si_{24}O_{87}$	4.760	6.128±0.005
		$(Fe_{54}Mg_1Si_3O_{10})(K_2O)_1$	6.025	6.567±0.009	$(Ca_2Mg_{30}Fe_4Al_2Si_{24}O_{87})(K_2O)_1$	4.800	6.369±0.006
		$(Fe_{54}Mg_1Si_3O_{10})(K_2O)_2$	6.036	7.062±0.009	$(Ca_2Mg_{30}Fe_4Al_2Si_{24}O_{87})(K_2O)_2$	4.838	6.612±0.006
		$(Fe_{54}Mg_1Si_3O_{10})(K_2O)_3$	6.055	7.503±0.013	$(Ca_2Mg_{30}Fe_4Al_2Si_{24}O_{87})(K_2O)_3$	4.870	6.853±0.005
		$(Fe_{54}Mg_1Si_3O_{10})(UO)_1$	6.062	6.155±0.011	$(Ca_2Mg_{30}Fe_4Al_2Si_{24}O_{87})(UO)_1$	4.806	6.202±0.006
		$(Fe_{54}Mg_1Si_3O_{10})(UO)_2$	6.095	6.265±0.009	$(Ca_2Mg_{30}Fe_4Al_2Si_{24}O_{87})(UO)_2$	4.849	6.273±0.007
		$(Fe_{54}Mg_1Si_3O_{10})(UO)_3$	6.132	6.345±0.010	$(Ca_2Mg_{30}Fe_4Al_2Si_{24}O_{87})(UO)_3$	4.890	6.322±0.007

Table S4. Comparison of Chemical potentials using different compositions. Calculated chemical potentials of K_2O and UO in $Fe_{54}Mg_1Si_3O_{10}$ and $Ca_2Mg_{30}Fe_4Al_2Si_{24}O_{87}$ compared with those in $Fe_{5.4}O$ and $MgSiO_3$ at 500 GPa and 9000 K.

P (GPa)	T (K)	System (i)	$\bar{\mu}_i^{Fe_{54}Mg_1Si_3O_{10}}$ (eV)	$\bar{\mu}_i^{Fe_{5.4}O}$ (eV)	$\bar{\mu}_i^{Ca_2Mg_{30}Fe_4Al_2Si_{24}Si_{24}O_{87}}$ (eV)	$\bar{\mu}_i^{MgSiO_3}$ (eV)
500	9000	K_2O	55.432±0.385	55.571±0.521	56.101±0.899	56.252±1.001
		UO	19.585±0.326	19.496±0.441	22.853±0.822	23.050±0.918

Table S5. Bader charges (δ) of K, Th, and U in metal ($Fe_{5.4}O$) and silicate ($MgSiO_3$) melts.

P (GPa)	*T* (K)	System (*i*)	$\delta_i^{Fe_{5.4}O}$	$\delta_i^{MgSiO_3}$
50	3500	K in K_2O	0.585±0.024	0.708±0.006
135	5000		0.350±0.030	0.634±0.012
500	9000		–0.080±0.036	0.350±0.032
1000	13000		–0.233±0.036	–0.043±0.094
50	3500	Th in ThO	1.652±0.156	2.577±0.042
135	5000		1.148±0.142	2.528±0.028
500	9000		0.875±0.112	2.100±0.140
1000	13000		0.681±0.108	1.358±0.308
50	3500	Th in ThO_2	1.991±0.114	2.532±0.014
135	5000		1.100±0.100	2.474±0.030
500	9000		0.857±0.074	2.061±0.098
1000	13000		0.736±0.088	1.508±0.158
50	3500	U in UO	1.558±0.164	2.468±0.036
135	5000		0.822±0.138	2.347±0.064
500	9000		0.421±0.110	1.661±0.132
1000	13000		0.285±0.064	1.052±0.218
50	3500	U in UO_2	1.839±0.104	2.441±0.052
135	5000		1.118±0.170	2.357±0.048
500	9000		0.432±0.098	1.809±0.182
1000	13000		0.238±0.068	1.135±0.286

Table S6. Comparison of Chemical potentials using different K-points. Calculated chemical potentials of K_2O, ThO_2, and UO in $Fe_{54}O_{10}$ using K-points 2×2×2 compared with those using Gamma point only at 50 GPa/3500 K and 1000 GPa/13000 K.

P (GPa)	T (K)	System (i)	$\bar{\mu}_i^{Fe_{5.4}O}$ (eV) (K-points 2×2×2)	$\bar{\mu}_i^{Fe_{5.4}O}$ (eV) (Gamma point only)
50	3500	K_2O	1.082±0.563	1.090±0.558
1000	13000		93.897±0.442	93.896±0.459
50	3500	ThO_2	–20.657±0.219	–20.637±0.229
1000	13000		71.838±0.148	71.840±0.156
50	3500	UO	–18.762±0.188	–18.741±0.200
1000	13000		51.212±0.457	51.234±0.459

Table S7. Key parameters in our models for the evolution of rocky exoplanets with Earth-like bulk compositions. The first six parameters were calculated as described in (*45*). We calculated G in this study to reproduce the adiabat shown in (*51*). We derived $T_{C,cold}$ using that adiabat, with $T_{C,warm}$ calculated following (*37*), as in (*44*).

Variable	Definition	Planet Mass (M_E)					
		1	2	3	4	5	6
R_P	Radius of the planet (km)	6371	7682	8571	9263	9839	10335
R_C	Radius of the core (km)	3301	3940	4343	4643	4884	5086
k_l	Thermal conductivity in the lower mantle (W/[m K])	9	11	13	15	17	20
r_l	Density in the lower mantle (kg/m^3)	5872	6547	7110	7602	8038	8441
g_l	Gravitational acceleration in the lower mantle (m/s^2)	11.9	16.7	20.6	24.0	27.1	29.9
P_C	Pressure at the core/mantle boundary (GPa)	144	273	408	546	683	822
Γ	Grüneisen-like parameter used to calculate the lower mantle temperature from the mantle potential temperature	1.11	1.11	1.09	1.06	1.04	1.01
$T_{C,cold}$	Temperature in the lower mantle when T_M = 2000 K (K)	2980	3362	3645	3848	4028	4147
$T_{C,warm}$	Mantle solidus temperature at the core/mantle boundary (K)	4429	6021	7302	8398	9351	10220
